# Mad28, a conserved actin-like protein in deep-branching magnetotactic bacteria, exhibits cell curvature-dependent localization

**DOI:** 10.1128/jb.00368-25

**Published:** 2025-11-24

**Authors:** Rino Shimoshige, Hirokazu Shimoshige, Azuma Taoka

**Affiliations:** 1 Graduate School of Natural Science and Technology, Kanazawa University 73674 , Kanazawa, Ishikawa, Japan; 2 Bio-Nano Electronics Research Center, Toyo University 133726 , Kawagoe, Saitama, Japan; 3 Institute for Extra-cutting-edge Science and Technology Avant-garde Research (X-star), Japan Agency for Marine-Earth Science and Technology (JAMSTEC) 13570, Yokosuka, Kanagawa, Japan; 4 Institute of Science and Engineering, Kanazawa University 73674 , Kanazawa, Ishikawa, Japan; 5 Nano Life Science Institute (WPI-NanoLSI), Kanazawa University 12858, Kanazawa, Ishikawa, Japan; University of Southern, Los Angeles, California, USA

**Keywords:** magnetotactic bacteria, live-cell imaging, bacterial organelles, cytoskeleton, actin-like protein

## Abstract

Magnetotactic bacteria (MTB) synthesize magnetic bacterial organelles called magnetosomes, which enable them to navigate along the geomagnetic field in aquatic environments. The actin-like cytoskeletal protein MamK forms filaments that associate with magnetosomes and mediate their positioning. Interestingly, in seven phyla, including *Desulfobacterota*, MTB encodes a second actin-like protein, Mad28, alongside MamK, within the magnetosome island—a genetic region responsible for magnetosome synthesis. In this study, we characterized the structure and function of this alternative magnetosome-associated cytoskeletal protein, Mad28. Magnetosome-specific localization of Mad28 in *Solidesulfovibrio magneticus* RS-1 was confirmed using immunoblotting, immunofluorescence microscopy, and correlative light and electron microscopy. To examine whether Mad28 and MamK have distinct or overlapping roles in magnetosome positioning, we tested the ability of Mad28^RS-1^ or MamK^RS-1^ to rescue the MamK-dependent static magnetosome positioning phenotype in *Magnetospirillum magneticum* AMB-1. Live-cell imaging revealed that MamK^RS-1^ expression restored static magnetosome positioning, whereas Mad28^RS-1^ expression had no effect, suggesting functional divergence between the two proteins. We further examined the potential role of Mad28 in sensing cellular geometry by comparing the localization of a Mad28-Dendra2 fusion protein in wild-type rod-shaped *Escherichia coli* and vibrio-shaped *E. coli* cells expressing Crescentin. Remarkably, Mad28 exhibited a curvature-dependent localization pattern in *E. coli*. These findings provide direct evidence that the actin-like protein Mad28 presents an affinity with membrane curvature in bacterial cells. In conclusion, the dual cytoskeletal systems—MamK and Mad28—contribute to magnetosome positioning through distinct mechanisms in deep-branching MTB.

**IMPORTANCE:**

Bacteria are capable of precisely positioning nanosized, membrane-enclosed organelles within their limited cellular spaces. This study shows that two distinct actin-like proteins contribute to magnetosome positioning through separate mechanisms in deep-branching magnetotactic bacteria. This contrasts with the evolutionary strategy observed in eukaryotic cells, where a single actin protein performs multiple functions. Furthermore, the findings suggest that the protein Mad28 is involved in sensing membrane curvature, introducing a novel functional property for bacterial actin-like proteins. These findings offer new insights into the role of the cytoskeleton in organelle positioning within micron-scale bacterial cells.

## INTRODUCTION

Cytoskeletons, once thought to be exclusive to eukaryotic cells, are now recognized as functional across all three life domains. In bacteria, a range of cytoskeletal elements, including actin-like cytoskeletal proteins, contribute to the spatial organization of intracellular components (1, 2). For example, MreB is essential for maintaining cell shape and organizing internal structures (3), while ParM facilitates the segregation of low-copy-number plasmids through dynamic filament assembly (4). Additionally, MamK forms filaments that align magnetosome chains along the cell axis in magnetotactic bacteria (MTB) (5, 6). Although eukaryotic cells coordinate organelle positioning and maintenance through complex networks of actin filaments, microtubules, and intermediate filaments, the mechanisms by which bacterial cells position their organelles using cytoskeletal systems remain much less understood.

Magnetosomes are well-characterized bacterial organelles synthesized in MTB that function as sensors of the Earth’s magnetic field, enabling magneto-aerotactic motility toward microaerobic or anaerobic environments suitable for their growth (7–11). Each magnetosome contains a single crystal of magnetite or greigite that mineralizes within a magnetosome membrane vesicle and aligns into a chain-like configuration within MTB cells. MTB represents a phylogenetically diverse group, exhibiting a wide range of morphological and physiological traits. Currently, MTB diversity has expanded to include representatives from 17 different bacterial phyla (12). All known MTB genomes encode magnetosome-associated proteins within a genomic region known as the magnetosome island (MAI) (13–15). The MAI contains *mam*, *mms*, *mad*, and *man* genes (16). Among them, nine genes—*mamA*, *mamB*, *mamE*, *mamI*, *mamK*, *mamM*, *mamO*, *mamP*, and *mamQ*—are conserved across most MTB genomes, regardless of whether the organisms produce magnetite or greigite and irrespective of their phylum. Conversely, many other genes found within the MAI are specific to particular phylogenetic groups.

The actin-like cytoskeletal protein MamK is one of the conserved proteins among MTB and polymerizes into double‐helical filaments that associate with the magnetosome chain (17–19). MamK function has been primarily studied in MTB model species of the genus *Magnetospirillum*, which belongs to the phylum *Pseudomonadota*. MamK maintains the proper positioning of the magnetosome chain. In *Magnetospirillum gryphiswaldense* MSR‐1, MamK repositions magnetosome chains from the new poles of daughter cells to the midcell region following cytokinesis (20). Conversely, in *Magnetospirillum magneticum* AMB-1, the MamK cytoskeleton anchors magnetosomes in a stable chain-like positioning, facilitating the formation of an effective magnetic dipole and preventing dispersion through simple diffusion (21).

Another actin-like protein, Mad28, is found within the MAIs of deep-branching MTB (22). The *mad* genes (Magnetosome‐associated *Deltaproteobacteria* or deep-branched) have been identified in MAIs belonging to the phyla *Desulfobacteriota*, *Nitrospirota*, *Bdellovibrionota*, *Omnitrophota*, *Fibrobacterota*, *Planctomycetota*, and *Rifleibacteriota* (16, 22–24), but are not conserved in the MTB of the phylum *Pseudomonadota*. To date, only a few studies have explored the function of Mad28. Awal et al. examined whether Mad28 from *Desulfamplus magnetovallimortis* BW-1 (phylum *Desulfobacteriota*), *Candidatus* Magnetobacterium bavaricum Mbar (phylum *Nitrospirota*), and *Nitrospirota* bacterium HCHbin1 (phylum *Nitrospirota*) could rescue the central magnetosome positioning defect in *mamK* mutants of *M. gryphiswaldense* MSR-1. They showed that Mad28 formed filamentous structures in MSR-1 cells and partially complemented the *mamK* mutant phenotypes (25). Additionally, they reported that Mad28 from BW-1 polymerized filamentous structure in the presence of ATP-gamma-S *in vitro* (25). In a separate study, Russell et al. analyzed phenotypes of mutants of *mad20*, *mad23*, *mad25*, and *mad26* (encoding coiled-coil proteins), as well as *mamK* and *mad28* (encoding actin-like proteins), in *Solidesulfovibrio magneticus* RS-1 (formerly *Desulfovibrio magneticus* RS-1) (26). The *mamK* and *mad28* mutant phenotypes differed. Although Δ*mad28* mutants retained the ability to form magnetosome chains, the chains were mislocalized away from the positively curved membrane regions where they are typically positioned in wild-type RS-1 cells. The authors proposed that Mad28 plays a role in localizing magnetosome chains along the positively curved (concave) membrane surfaces. Deep-branching MTB typically possesses the two magnetosome-associated cytoskeletal proteins, MamK and Mad28. Determining whether these actin-like proteins have complementary or distinct functions is crucial for understanding the contributions of bacterial cytoskeletal systems to organelle positioning and functional specialization.

In this study, we aimed to characterize Mad28 from *S. magneticus* RS-1. RS-1 is a freshwater, anaerobic, sulfate-reducing magnetotactic bacterium, which belongs to the phylum *Desulfobacteriota* isolated in 1993 by Sakaguchi et al. (27–29). It biomineralizes a small number (ca. six per cell) of bullet-shaped magnetite crystals, which align as a single magnetosome chain along the central region of the concave face of the vibrio-like cell (29). As an MTB species phylogenetically related to RS-1, we used *Fundidesulfovibrio magnetotacticus* FSS-1 (FSS-1). FSS-1 is a freshwater, strictly anaerobic, sulfate-reducing MTB belonging to the phylum *Desulfobacteriota*, which was isolated and characterized by Shimoshige et al. in 2021 (30). The *mad28* gene is also conserved in the FSS-1 genome. Although most genetic and biochemical studies have focused on Alphaproteobacterial MTB, RS-1 is the best-studied MTB outside the phylum *Pseudomonadota*. Genome analysis (31) and proteomic studies (32) of RS-1 have been previously conducted, and a targeted mutagenesis system has been developed for this strain (33). Notably, cryo-ultramicrotomy and cryo-electron tomography analyses of RS-1 cellular ultrastructure revealed that its magnetite crystals are not enclosed by membranes (34). On the other hand, the transmission electron microscopy (TEM) images of purified RS-1 magnetosomes revealed an amorphous shell-like contrast surrounding each crystal, which may represent remnants of the membrane that remained after extraction from the cells (35). Based on observations of RS-1 cells, in which a comprehensive series of deletion mutants for each *mad* gene in MAI, Russell et al. (26) proposed a unique biomineralization process mediated by the Mad proteins. These results suggest that the magnetosome formation process differs between AMB-1/MSR-1 and RS-1. Furthermore, another iron-rich bacterial organelle—the ferrosome—was identified in RS-1, and its biosynthetic genes (*fez* genes) were also detected (36).

In this study, we analyzed the localization of Mad28 in RS-1 cells using an immunohistochemical approach and correlative light and electron microscopy (CLEM). Furthermore, we determined whether Mad28 and MamK from RS-1 could functionally rescue dynamic magnetosome positioning in AMB-1. Additionally, we examined the effects of altered cellular morphology on Mad28 localization using *Escherichia coli* cells with Crescentin (CreS)-induced curvature.

## RESULTS

### Mad28 expression in *S. magneticus* RS-1 and *Fundidesulfovibrio magnetotacticus* FSS-1

The *mad28* genes were recently identified in the genomes of deep-branching MTB as encoding previously uncharacterized bacterial actin-like proteins. To the best of our knowledge, the subcellular localization of Mad28 has not yet been explored. In this study, we examined the expression and localization of Mad28 in *S. magneticus* RS-1 (RS-1) and *F. magnetotacticus* FSS-1 (FSS-1) cells, which is a sulfate-reducing magnetotactic bacterium belonging to the same phylum as RS-1. We obtained polyclonal rabbit anti-Mad28 antibodies raised against recombinant His-tagged Mad28 from RS-1, which was used as the antigen (Fig. S1A; see Materials and Methods).

**Fig 1:**
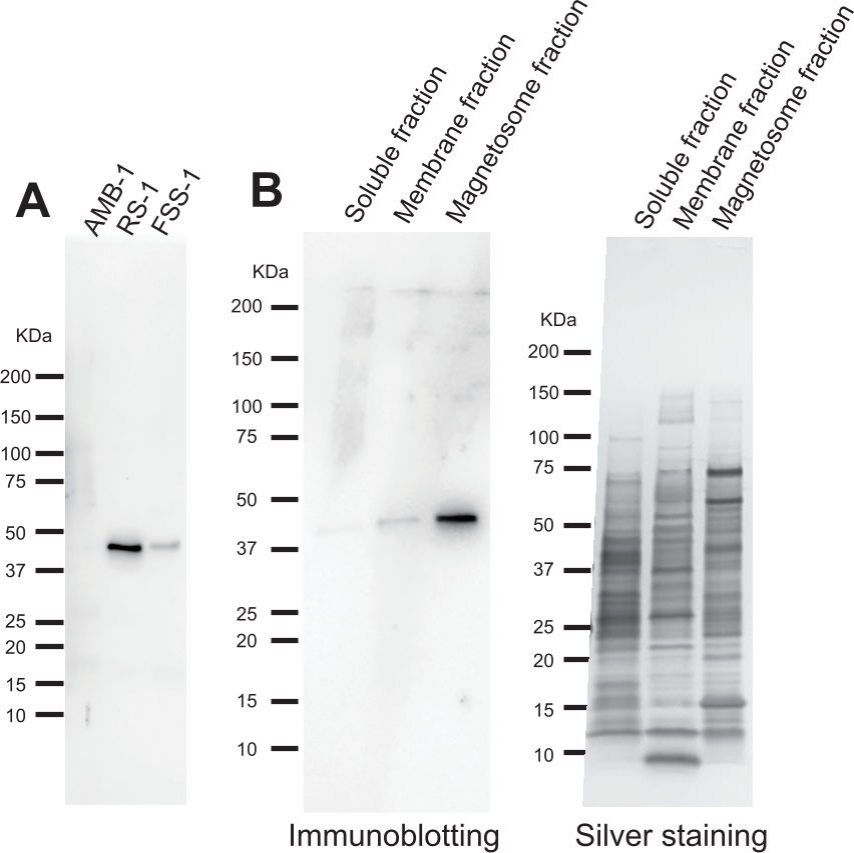
Mad28 expression in RS-1 and FSS-1. (**A**) Immunoblot analysis using anti-Mad28^RS-1^ antibodies in *M. magneticum* AMB-1, *S. magneticus* RS-1, and *F. magnetotacticus* FSS-1 lysates. Proteins (7.5 µg per lane) extracted from the respective cells were loaded into each lane. The antibodies generated against Mad28 exhibited monospecificity, as indicated by single positive bands corresponding to an apparent molecular mass of 42 kDa. (**B**) Immunoblot (left) and SDS-PAGE (right) analyses of the soluble, membrane, and magnetosome fractions from *S. magneticus* RS-1 cells. Proteins (0.6 µg per lane) were loaded into each lane. Molecular mass markers (Precision Plus Protein Standards; Bio-Rad) are indicated on the left side of the blots and gels.

We examined the expression of Mad28 in RS-1 and FSS-1 cell extracts using immunoblotting (Fig. 1A). A single positive band observed at approximately 49.8 kDa corresponded to the predicted molecular mass of Mad28 (42.6 kDa), as encoded by the *mad28* genes of RS-1 and FSS-1, indicating that Mad28 is natively expressed in both species. This finding provides the first evidence of Mad28 protein expression in *Desulfobacteriota* MTB. The anti-Mad28^RS-1^ antibody cross-reacted with Mad28^FSS-1^ (68.8% identity and 83.1% homology between Mad28^RS-1^ and Mad28^FSS-1^). No positive bands were detected in *M. magneticum* AMB-1 (AMB-1) cell extracts (negative control), indicating the specificity of the anti-Mad28 antibodies for the *Desulfovibrionaceae* family.

Subsequently, we analyzed the subcellular localization of Mad28 in RS-1 using immunoblots of cell fractions, including soluble, membrane, and magnetosome fractions (Fig. 1B). The Mad28-positive band was most intense in the magnetosome fraction, despite equal amounts of total protein being loaded in each lane, suggesting that Mad28 is specifically localized in magnetosomes. A faint positive band was also observed in the membrane fraction (Fig. 1B).

### Mad28 localizations in RS-1 and FSS-1 cells

**Fig 2:**
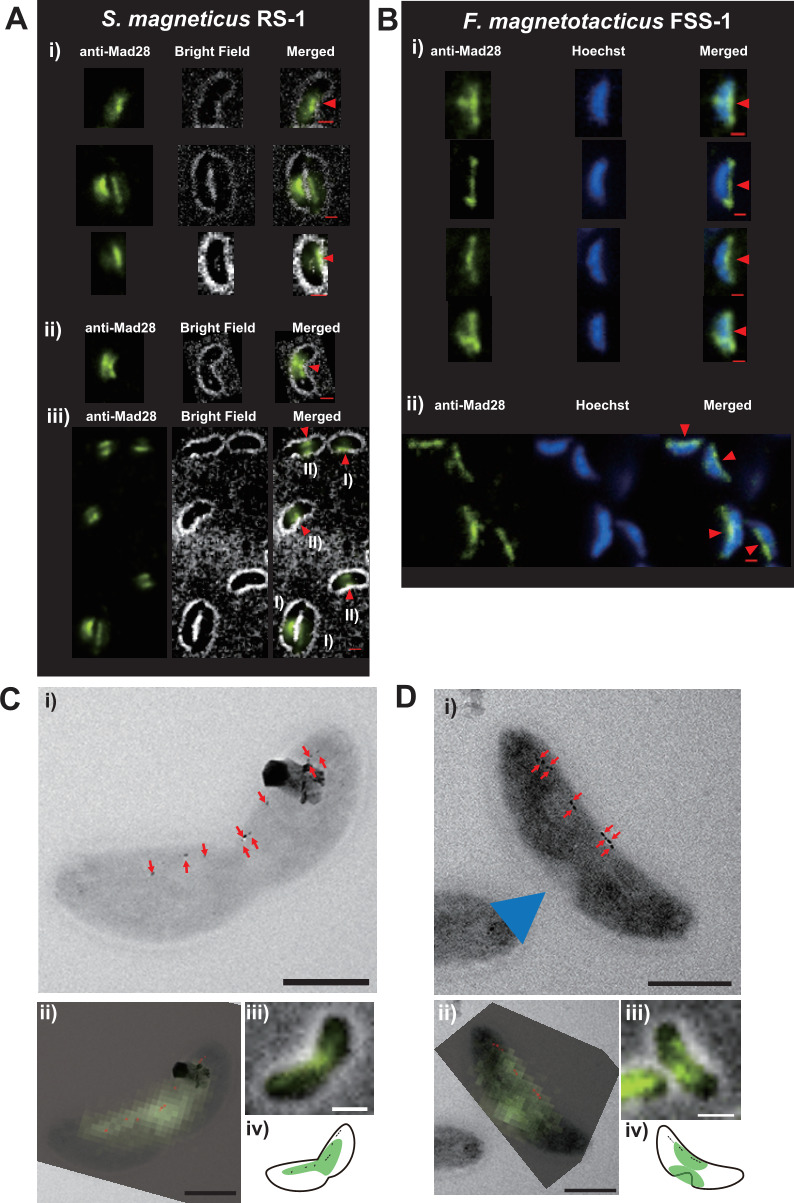
Mad28 localizations using immunohistochemistry. (**A**) Immunofluorescence staining of RS-1 cells using anti-Mad28^RS-1^ antibodies. Two distinct Mad28 localization patterns—Pattern I and Pattern II—were observed. (i) RS-1 cells exhibiting Pattern I, characterized by a single band structure located along the concave face of the cells. (ii) An RS-1 cell exhibiting Pattern II, characterized by two bands or a belt-like structure. (iii) Low-magnification image of immunofluorescence-stained RS-1 cells. In Panels (iii), “(I)” and “(II)” illustrate cells with Pattern I and Pattern II Mad28 localization, respectively. Red arrowheads indicate the concave face of each cell. (**B**) Immunofluorescence staining of FSS-1 cells using anti-Mad28^RS-1^ antibodies. Hoechst staining was used to visualize cell bodies. (i) In FSS-1 cells, Mad28 was localized to the concave face, appearing as a single, thin, fluorescent signal. (ii) Low-magnification image of the immunofluorescence-stained FSS-1 cells. (**C, D**) CLEM of RS-1 cells exhibiting Patterns I (**C**) and Pattern II (**D**) Mad28 localization. (i) TEM images of RS-1 cells; red arrows indicate magnetite crystals, while blue arrowheads denote constriction sites. (ii) CLEM images with magnetite crystals colored in red. (iii) Immunofluorescence images superimposed on bright-field images. (iv) Schematic images of the CLEM images. Scale bars: 1 µm.

The subcellular localization of Mad28 was examined in RS-1 and FSS-1 cells using immunofluorescence microscopy (IFM). Cells in the late logarithmic growth phase were used for analysis. Fig. 2A shows an IFM image of RS-1, with two distinct Mad28 localization patterns observed. Approximately 46% of RS-1 cells exhibited a single, thin, band-like Mad28 signal along the positively curved face at the midcell region (Fig. 2Ai; Pattern I), while approximately 54% showed a double-band or belt-like signal at the midcell region (Fig. 2Aii; Pattern II). In FSS-1 cells, Mad28 also formed filamentous structures along the positively curved face but showed only a single localization pattern (Fig. 2Bi). The Mad28 signal extended to both cell poles. Due to the low contrast of bright-field images of FSS-1, Hoechst staining was used to visualize the cell bodies. Nearly all RS-1 and FSS-1 cells exhibited positive Mad28 signals (Fig. 2Aiii and Bii; Fig. S2 and S3). Conversely, no signals were detected in IFM experiments using serum from preimmune rabbits (Fig. S2 and S3).

To further examine the localization of Mad28 relative to magnetosomes in RS-1 cells, CLEM was employed. Chemically fixed RS-1 cells were mounted on grids, followed by immunofluorescence staining and observation using both fluorescence microscopy and transmission electron microscopy (TEM). RS-1 cells exhibit a vibrioid morphology, characterized by distinct concave and convex faces. TEM revealed that the magnetosomes, composed of bullet-shaped magnetite crystals, were localized to the concave face (Fig. 2Ci and Di). In cells exhibiting Pattern I, the Mad28 fluorescence signals broadly coincided with magnetosome localization, suggesting that a substantial portion of Mad28 localizes to the magnetosome chain (Fig. 2C; Fig. S4A). These signals were localized in the central, highly curved region of the concave face of the cells.

In cells exhibiting Pattern II, although the magnetosomes remained on the concave face, Mad28 signals were detected on both the concave and convex faces in the midcell region. Notably, the convex face of these cells exhibited pronounced constriction, likely representing a future septation site during cell division (Fig. 2D and S4B). This pattern may be indicative of cell division-associated processes. The heterogeneity of Mad28 localization in RS-1 suggests that its positioning dynamically changes throughout the cell cycle.

### Mad28 has a distinct cytoskeletal function from MamK

Immunoblotting and immunofluorescence staining revealed that a portion of Mad28 localizes to the magnetosomes. In RS-1, two actin-like proteins—Mad28 and MamK—are encoded within the MAI. It remains unclear whether these two proteins have redundant or distinct functions in magnetosome synthesis. Previously, Awal et al. reported that Mad28 from *Desulfamplus magnetovallimortis* BW-1 (phylum *Desulfobacteriota*) could functionally rescue midcell positioning of the magnetosome chain in a Δ*mamK* mutant of *M. gryphiswaldense* MSR-1 (25). In that study, magnetosome positioning in MSR-1 cells was analyzed using TEM during rescue experiments. In this study, we examined whether Mad28 and MamK from RS-1 could rescue the static magnetosome-positioning phenotype of the Δ*mamK* mutant of *M. magneticum* AMB-1 using live-cell imaging of magnetosome dynamics. In AMB-1, MamK anchors magnetosomes into a static patch-like arrangement (21). Conversely, magnetosomes become dispersed in the AMB-1 Δ*mamK* mutant due to passive diffusion in the cells (21).

To visualize magnetosome dynamics in living cells, time-lapse images were taken every minute using bright-field and GFP fluorescence through HILO microscopy. As controls, Fig. S5 presents the dynamics of magnetosomes in Δ*mamK* mutants (Fig. S5A) and in MamK-rescued cells (Fig. S5B), where MamK^AMB-1^ was expressed from a plasmid in the Δ*mamK* mutant. In the time-lapse still images, the MamC-GFP signals were rainbow-colored sequentially from red to blue over a 14-minute observation period (Fig. S5iii). These colored images were superimposed to create a single composite image of all the different colored time-lapse still images (Fig. S5iv), enabling visualization of magnetosome motility. In the merged images, white spots indicate static magnetosomes, whereas colored spots represent dynamic magnetosomes. In the Δ*mamK* mutants, magnetosomes appeared as colored spots in the merged colored image, indicating dynamic behavior (Fig. S5A). Conversely, in the MamK-rescued cells, magnetosomes appeared as white spots throughout the 14-minute observation, reflecting a static arrangement (Fig. S5B).

**Fig 3:**
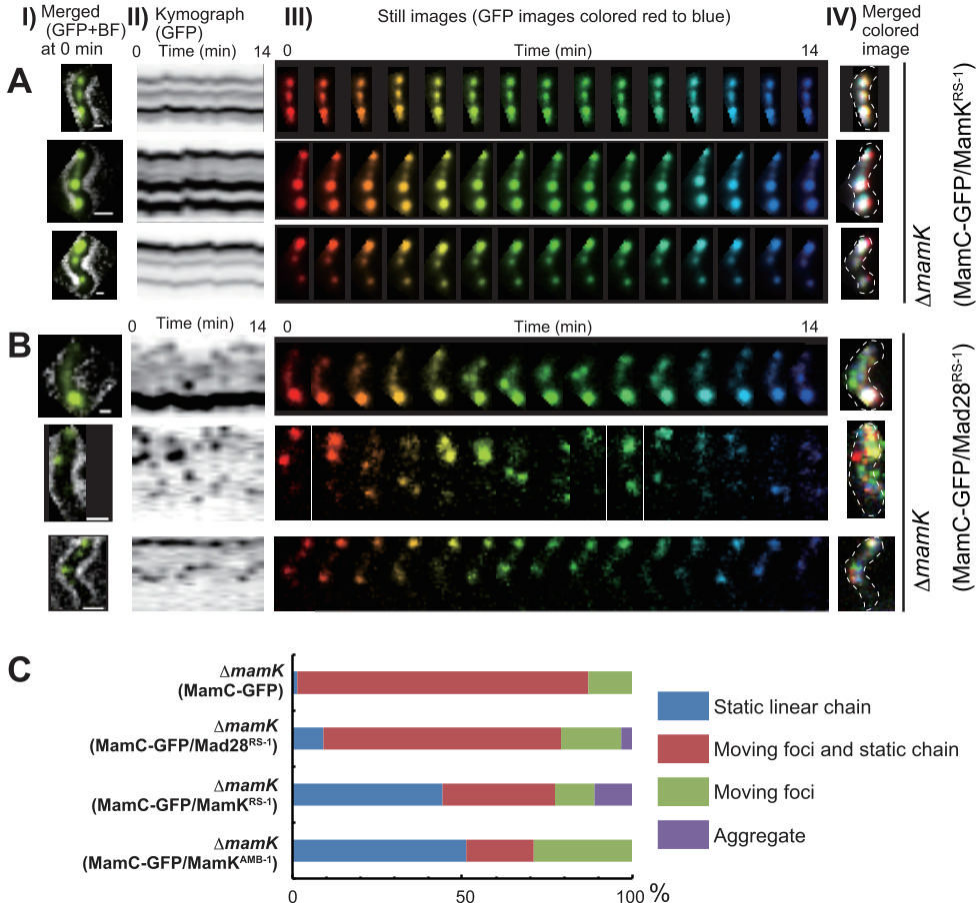
Rescue of MamK-dependent static magnetosome positioning. Magnetosomes were visualized using MamC-GFP in the *mamK* deletion strain AMB-1 expressing MamK^RS-1^ (**A**) and Mad28^RS-1^ (**B**). (Column I): Merged GFP and bright-field images of cells at time zero. Scale bars: 1 µm. (Column II): Kymographs of GFP trajectories generated in maximum projections. (Column III): Time-lapse still images acquired over a 14-minute interval, sequentially rainbow colored from red to blue. (Column IV): Merged images of the rainbow-colored time-lapse still images shown in column III. White signals indicate static GFP fluorescence, while colored signals denote dynamic GFP fluorescence. These images indicate that magnetosomes remained static in Δ*mamK* cells expressing MamK^RS-1^ but were dynamic in cells expressing Mad28^RS-1^. (**C**) Dynamic localization patterns of magnetosomes. Magnetosome dynamics were assessed through 1-hour time-lapse observations in Δ*mamK* AMB-1 cells (*n* = 143), Mad28^RS-1^-expressing Δ*mamK* cells (*n* = 220), MamK^RS-1^-expressing Δ*mamK* cells (*n* = 172), and MamK^AMB-1^-complemented Δ*mamK* cells (*n* = 207). In Mad28^RS-1^-expressing Δ*mamK* AMB-1 cells, MamC-GFP-labeled magnetosomes exhibited partial motility, whereas those in MamK^RS-1^ or MamK^AMB-1^-complemented cells remained predominantly static.

We coexpressed MamK^RS-1^ and MamC-GFP (Fig. 3A) or Mad28 ^RS-1^ with MamC-GFP (Fig. 3B) in ∆*mamK* AMB-1 cells. In MamK^RS-1^-expressing Δ*mamK* cells, magnetosomes appeared as white spots in the merged colored time-lapse images, similar to those observed in MamK^AMB-1^-rescued cells (Fig. 3A; Movie S1). Moreover, the kymographs—depicting the trajectory of magnetosomes in a cell during the 14-minute observation—showed parallel lines, indicating static magnetosome positioning. Conversely, in Mad28^RS-1^-expressing Δ*mamK* cells, magnetosomes appeared as multicolored spots in the merged colored time-lapse images (Fig. 3B; Movie S2), and the corresponding kymographs showed discontinuous signals, indicating dynamic magnetosome movement. A 1-hour time-lapse movie revealed that MamK^RS-1^-expressing Δ*mamK* cells maintained magnetosomes as static, linear spots (Movie S3), while most Mad28^RS-1^-expressing Δ*mamK* cells failed to anchor magnetosomes at fixed positions (Movie S4). Fig. 3C illustrates the distribution of magnetosome dynamics patterns per cell. We classified these patterns as “static linear chain,” “moving foci and static chain,” “moving foci,” and “aggregation.” Nearly all Δ*mamK* cells (99%) contained moving magnetosomes, while 51% of MamK^AMB-1^-rescued Δ*mamK* cells had magnetosomes positioned in static linear chains, consistent with a previous complementary study (21). In MamK^RS-1^-expressing Δ*mamK* time-lapse movies, 44% exhibited static linear magnetosomes, indicating that heterologous MamK partially rescued magnetosome static positioning in AMB-1. Interestingly, only 9% of Mad28^RS-1^-expressing Δ*mamK* cells exhibited static linear positioning, while 87% showed moving magnetosomes. These results indicate that Mad28 is able to partially compensate for the role of MamK anchoring magnetosomes along the long axis at the midcell in AMB-1. Expression of Mad28 from the coexpression plasmid used in the live-cell imaging experiments was confirmed in ∆*mamK* AMB-1 cells (Fig. S1B). This complementation test underscores a functional divergence between MamK and Mad28 from RS-1 magnetosome positioning in AMB-1.

### Heterologous expressions of Mad28 in rod-shaped *E. coli* and spiral-shaped AMB-1

The MamK complementation test suggested that Mad28 functions differently from MamK. In a previous study, Russell et al. proposed that Mad28 aligns magnetosomes with positive curvature early in chain formation, based on TEM observations of *mad28* deletion in RS-1 mutant cells (26). We previously showed that Mad28 localizes to the concave face of RS-1 cells (Fig. 2), raising the possibility that Mad28 enables the sensing of membrane curvature and preferentially localizes to positively curved regions. To evaluate this possibility, we examined whether the alteration of Mad28 localization was influenced by cell morphology. We heterologously expressed Mad28 fused to Dendra2 in rod-shaped *E. coli* and spiral-shaped AMB-1. Dendra2 is a monomeric photoconvertible green fluorescence protein from octocoral (37). In *E. coli*, the C-terminal fusion Mad28-Dendra2 formed filamentous structures, whereas the N-terminal fusion Dendra2-Mad28 was cytosolic and did not form filamentous structures (Fig. S6). Therefore, Mad28-Dendra2 was used in subsequent experiments.

**Fig 4:**
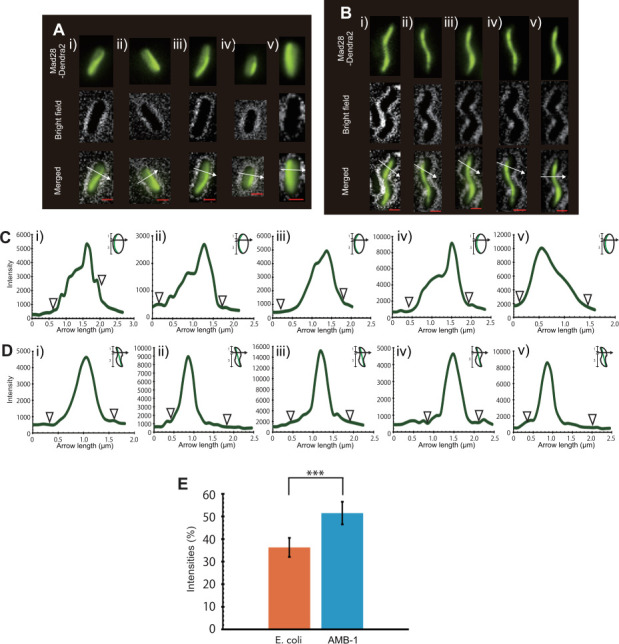
Heterologous expression of Mad28^RS-1^ in BL21(DE3) and AMB-1. (**A, B**) Mad28^RS-1^-Dendra2 expressed in BL21(DE3) (**A**) and AMB-1 (**B**). White arrows indicate the direction and position of fluorescence intensity measurements. Scale bars: 1 µm. (**C, D**) Fluorescence intensity profiles of Mad28^RS-1^-Dendra2 in BL21(DE3) (**C**) and AMB-1 (**D**) cells, measured along the white arrows. White arrowheads show the edges of the cells. (**E**) Proportion intensities of three pixels corresponding to the peak tips in the Mad28 localization line profiles (*P* < 0.0001).

Fig. 4A and B show the localization patterns of Mad28-Dendra2 in *E. coli* and AMB-1, respectively. In both organisms, Mad28 formed filamentous structures aligned along the long axis of the cells, suggesting that it can polymerize into filaments without other elements from RS-1. However, the localization patterns differed between the two organisms. In AMB-1, Mad28 formed a sharp filamentous structure extending from pole to pole and was positioned along the concave cell surface. Conversely, in *E. coli*, Mad28 appeared as diffuse, shapeless filamentous structures positioned along the long axis of the cells. Fig. 4C and D present transverse line profiles of the Dendra2 signal at one-third the length of the long axis of the cell in *E. coli* and AMB-1, respectively. In *E. coli*, the Mad28 signal was broadly distributed across the cell width (Fig. 4C), while in AMB-1, it exhibited sharp, discrete peaks at specific positions (Fig. 4D). Figure 4E presents the percentage intensities of three peak pixels from the line profiles of 50 cells. The peak intensities were significantly higher in AMB-1 than in *E. coli* (*P* < 0.0001), indicating that Mad28 forms more defined filamentous structures in AMB-1. These findings suggest that cell morphology affects Mad28 localization. However, differences in the expression levels of Mad28 between AMB-1 and *E. coli* may also influence these localization patterns. To further clarify whether cell shape alone affects Mad28 localization, we next altered the *E. coli* morphology.

### Mad28 exhibits curvature-dependent localization

CreS, a bacterial intermediate filament-like protein encoded by *Caulobacter vibroides* (formerly *C. crescentus*), can induce cell curvature, resulting in a vibrio-shaped cell shape with distinct concave and convex faces (38). To test the hypothesis that Mad28 shows an affinity for membrane curvature, we examined its localization in rod- and vibrio-shaped *E. coli* cells. CreS and Mad28-Dendra2 were coexpressed from compatible plasmids, pET15b-CreS (pMB1 ori) and pBBR111-mad28-Dendra2 (pBBR ori). To induce vibrio-like morphology, CreS-expressing cells were grown slowly in a glycerol-containing M9 medium supplemented with cephalexin to inhibit cell division. Control rod-shaped cells (lacking pET15b-CreS) were grown under similar conditions.

**Fig 5:**
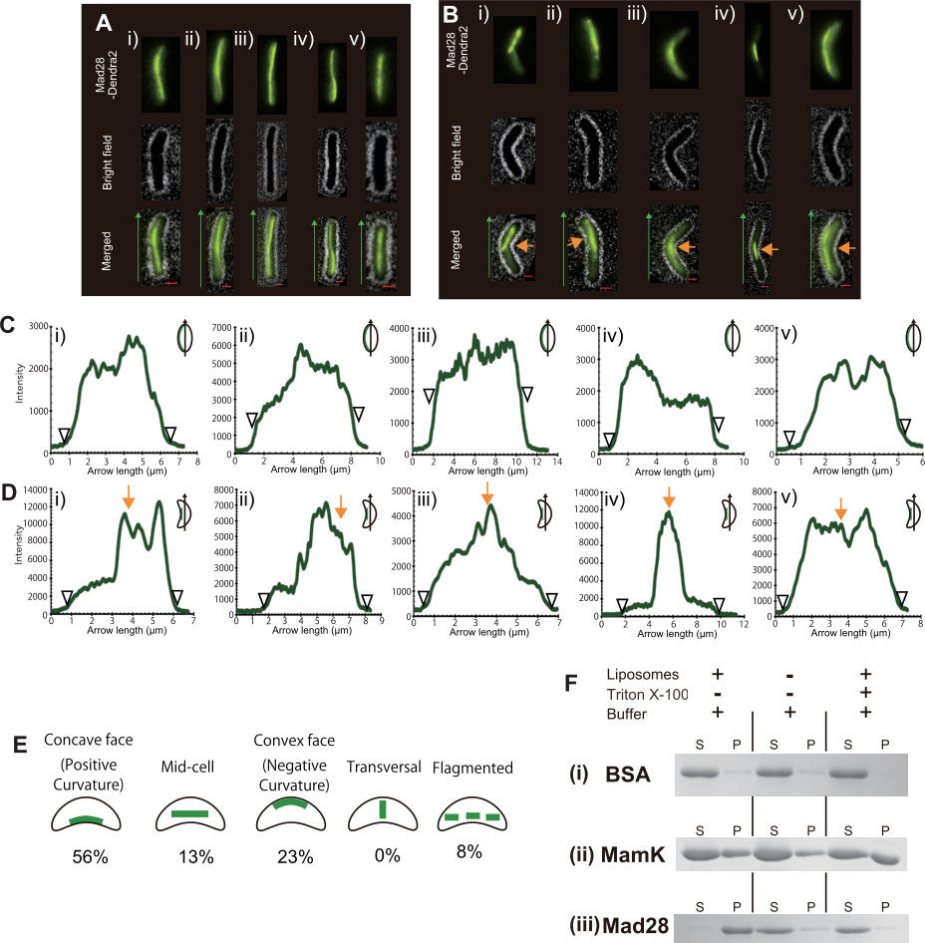
Heterologous expression of Mad28^RS-1^ in rod- and vibrio-shaped *E. coli* (HMS174). (**A, B**) Heterologous expression of Mad28^RS-1^ in rod-shaped (**A**) and vibrio-shaped (**B**) *E. coli* (HMS174). Green arrows indicate the direction and position of fluorescence intensity measurements. Scale bars: 1 µm. (**C, D**) Fluorescence intensity profiles of Mad28^RS-1^-Dendra2 in rod-shaped (**C**) and vibrio-shaped (**D**) *E. coli,* measured along the green arrows. Orange arrows indicate the concave region. (**E**) Proportion of Mad28 localization positioning in cells. Cells were classified into five phenotypes based on the observed positioning of the Mad28 filamentous structure near the concave region. Concave face: Mad28 filamentous structure adjacent to the concave membrane. Midcell: Mad28 filamentous structure positioned in the middle of the cell. Convex face: Mad28 filamentous structure adjacent to the convex membrane. Transversal: Mad28 filamentous structure localized along the short axis of the cell. Fragmented: Mad28 formed multiple short filamentous structures within the cell. (**F**) Pelleting assay with artificial liposomes. Coprecipitation of BSA (i), MamK^AMB-1^ (ii), and Mad28 (iii) was tested using artificial liposomes generated from *E. coli* polar lipids. In the presence of liposomes, BSA and MamK did not precipitate significantly, while Mad28 was detected in the pellet fraction (P). Conversely, Mad28 was detected in the supernatants (S) when incubated without liposomes or with liposomes plus the detergent Triton X-100.

In the rod-shaped cells, Mad28 formed a long filamentous structure extending from pole to pole along the long axis of the cells (Fig. 5A). Conversely, in the vibrio-like shaped cells, Mad28 formed a shorter filamentous structure localized around the curved (bent) region of the cells (Fig. 5B). The Mad28-Dendra2 expression levels in both rod- and vibrio-shaped cells were comparable (Fig. S1C and D). Transverse line profiles of Mad28 localization in both cell types were compared (Fig. S7); however, there was no significant difference in the sharpness of the peak intensities between the two morphologies. Fig. 5C and D show longitudinal line profiles of Mad28 signals along the cell length. In rod-shaped cells, Mad28 was distributed uniformly from pole to pole, while in vibrio-shaped cells, Mad28 was localized around the bent region (as indicated by orange arrows). To determine whether cell morphology also affected the localization of MamK^RS-1^, we examined MamK distribution under similar conditions (Fig. S8). MamK formed long, continuous, rope-like filamentous structures distributed gently along the cell periphery in both rod- and vibrio-shaped cells. The localization of MamK filaments did not correlate with curvature at the bent region. These results indicate that high cell curvature alters Mad28 localization in *E. coli*, highlighting a functional difference between Mad28 and MamK.

Notably, approximately 56% of Mad28 filamentous structures were positioned at the concave face (positive curvature) of the bent region in vibrio-shaped cells (Fig. 5E), suggesting that Mad28 may have an affinity with membrane curvature and preferentially localize to positively curved regions of the inner cytoplasmic membrane surface.

### Mad28 can bind to the bacterial membrane

Fig. 5 shows that the vibrio-shaped cellular morphology influences the localization of the Mad28 filamentous structure. This observation suggests that Mad28 may interact with the inner membrane and have an affinity for membrane curvature. To examine this, we tested whether Mad28 could attach to a lipid membrane (Fig. 5F). Purified Mad28 was mixed with artificial liposomes, prepared from *E. coli* polar lipids, incubated for 1 h, and then subjected to ultracentrifugation to assess coprecipitation with the liposomes. Although bovine serum albumin (BSA) and MamK^AMB-1^ did not co-precipitate with the liposomes, Mad28 was primarily detected in the pellet fraction. When 1% Triton X-100 was added to the mixture to solubilize the liposomes, the Mad28 band appeared in the supernatant, indicating that Mad28 was not aggregated but was associated with the liposomes. These results show that Mad28 can interact with the lipid membrane surface, despite lacking a transmembrane region. Thus, Mad28 is a membrane-associated actin-like protein.

## DISCUSSION

Bacterial cell morphology, shaped by the peptidoglycan layer, exhibits a geometrically ordered structure. The interplay between membrane curvature-sensing proteins and magnetosome positioning proteins suggests that bacterial cells utilize this ordered morphology as a spatial cue for accurate organelle placement. Using live-cell fluorescence imaging, this study shows that the actin-like protein Mad28 is positioned according to cellular curvature, indicating that Mad28 has an affinity with membrane curvature and associates with the concave side of the cell. A comparative analysis of Mad28 localization in both rod-shaped and vibrio-shaped *E. coli* cells supports a curvature-dependent mechanism, with Mad28 preferentially associated with positive curvature regions, particularly at the concave cell surface (Fig. 5). This finding is corroborated by quantitative line profile analyses and fluorescence intensity measurements, which reveal sharper and more localized filament formation in spiral-shaped AMB-1 compared to the more diffuse pattern observed in *E. coli* (Fig. 4).

The function of Mad28 appears similar to that of MamY in *Magnetospirillum gryphiswaldense* MSR-1 cells. Previous studies have shown that MamY forms a filamentous structure resembling a cytoskeleton and acts as a curvature-sensing protein (39), playing a vital role in the spatial organization of magnetosomes. Therefore, magnetosome positioning is likely regulated by a combination of two cytoskeletal components: MamK, which dynamically repositions magnetosomes and anchors them to prevent random diffusion, and a curvature-sensing cytoskeletal element. In deep-branching MTB, Mad28 likely contributes to magnetosome positioning in response to membrane curvature.

IFM revealed two patterns of Mad28 localization in RS-1 cells: a single central band-like pattern (Pattern I) and a double-band or belt-like pattern (Pattern II) (Fig. 2; Fig. S4). In Fig. 1B, a portion of Mad28 protein was detected in the membrane fraction. The CLEM images of RS-1 cells exhibiting Pattern II in Fig. 2D revealed that some Mad28 signals were localized on the convex surface, apart from the magnetosome positions, suggesting that not all Mad28 proteins were associated with magnetosomes. Instead, a subset of Mad28 appeared to be localized on the convex surface of the cytoplasmic membrane in Pattern II cells. The RS-1 cells used to prepare the lysate for the immunoblotting shown in Fig. 1B likely included a fraction of these Pattern II cells. Therefore, the detection of Mad28 in the membrane fraction in Fig. 1B is reasonable.

In Pattern II, Mad28 was observed on both the concave and convex faces in the midcell region. Given that CLEM images of Pattern II cells appear to reflect cell division due to septum formation, this pattern may be associated with cytokines (Fig. 2D; Fig. S4B). Therefore, the curvature alteration caused by cell division might drive double-band-like localization. However, live-cell imaging is necessary to observe the intracellular dynamics of magnetosomes and Mad28 to confirm this possibility. In Pattern I cells, CLEM images show that the Mad28 fluorescence signal largely overlaps with the position of magnetite crystals (Fig. 2C; Fig. S4A), whereas in Pattern II cells, such overlap is not observed (Fig. 2D; Fig. S4B). In Pattern II, Mad28 is localized in the midcell region, while the magnetite crystals are positioned in the cell’s positively curved regions. If Mad28 repositioning occurs during cell division, its midcell localization might contribute to *de novo* magnetosome formation following cytokinesis. Russell et al. previously reported that Mad28 is primarily localized in the cell lysate during the early stages of magnetosome formation (26), which is consistent with our CLEM results. Conversely, in FSS-1 cells, Mad28 was uniformly localized to the concave face (Fig. 2B; Fig. S3). This difference may be attributed to variations in the growth stage between RS-1 and FSS-1 cells used for immunofluorescence staining. Specifically, RS-1 cells were harvested during the early stationary phase (3 days of cultivation), while FSS-1 cells were collected at the late stationary phase (10 days of cultivation), reflecting their slower growth rate.

Earlier, Awal et al. reported that Mad28 from BW-1 could effectively restore the mid-cell positioning of magnetosome chains in the Δ*mamK* mutant cells of MSR-1 (25). However, in this study, Mad28^RS-1^ was only able to partially rescue magnetosome positioning in the Δ*mamK* mutant cells of AMB-1. The similarity and identity of amino acid sequences between Mad28^RS-1^ and Mad28^BW-1^ are 72.5% and 53.7%, respectively. Structural modeling using AlphaFold2 indicates that the subdomain structures of Mad28 from RS-1, BW-1, and other strains are well conserved (Fig. S9A). Additionally, residues characteristic of the actin superfamily are also conserved in Mad28 proteins (Fig. S9B). This conservation suggests that the characteristics and functions of Mad28 proteins are likely similar across these strains, which raises questions about the apparent discrepancy between the two rescue experiments. The difference in results may stem from the distinct phenotypes used in each experiment. The previous study involving MSR-1 assessed whether magnetosome mid-cell positioning was restored, while the current study with AMB-1 evaluated whether magnetosome static anchoring was recovered. These different assessments may reflect various aspects of MamK cytoskeletal function in magnetosome positioning. Therefore, the discrepancy in results may arise from differences in the extent and nature of the MamK functions that Mad28 can complement in MSR-1 and AMB-1.

According to structural modeling with AlphaFold2, Mad28 conserves at least one amphipathic α-helix (Fig. S9C). In *Bacillus subtilis,* SpoVM proteins that contain amphipathic α-helices insert into membranes through their hydrophobic face and specifically localize to the positively curved membranes of the forespore while avoiding the negatively curved cytoplasmic membrane (40–42). This curvature-sensing ability is mediated by the amphipathic α-helix of SpoVM (42). To explore whether the N-terminal amphipathic α-helices of Mad28 are involved in membrane curvature sensing, we examined the subcellular localization of N-terminally truncated Mad28 variants—Mad28∆1-30, Mad28∆1-48, and Mad28∆1-90— in *E. coli* and AMB-1. These Mad28 mutants lack the first amphipathic α-helix, the entire N-terminal region, and the whole N-terminal region with part of the actin-like structure, respectively. None of the mutant Mad28 proteins formed filamentous structures in either *E. coli* or AMB-1 (Fig. S10). Although this study did not elucidate the precise mechanism by which Mad28 presents an affinity with membrane curvature, further exploration of its biophysical properties is essential to clarify the molecular basis of membrane association and polymerization behavior of Mad28.

In this study, we showed that cell morphology influences Mad28 localization through heterologous expression in rod-shaped and vibrio-like-shaped *E. coli* cells. These findings suggest that Mad28 is a novel type of bacterial cytoskeletal element comprising a membrane curvature-sensing actin-like protein. This curvature-sensing behavior resembles that of another membrane-associated protein, MamY, indicating a potentially conserved mechanism among diverse MTB for magnetosome positioning. The interplay between dynamic repositioning and static anchoring, mediated by MamK, and the curvature-dependent positioning mediated by Mad28 provides new insights into bacterial cytoskeletal adaptation and the molecular mechanisms that regulate organelle positioning in prokaryotic cells.

## MATERIALS AND METHODS

### Cultivations

*M. magneticum* AMB-1 and AMB-1–derived strains were cultured in a chemically defined liquid medium (MG medium) at 28°C in the dark (43). The *E. coli* strains used in this study—wild-type, BL21(DE3), and HMS174—were cultivated in LB broth (44) at 37°C unless otherwise specified. *S. magneticus* RS-1 strain was cultured at 28°C in RS-1 growth medium, as previously described (34). RS-1 cultivation was performed anaerobically in test tubes sealed with butyl rubber stoppers (Sanshin). Each test tube contained 21 mL of medium, which was bubbled twice with nitrogen gas for 10 min, using a long injection needle, and then autoclaved. Anaerobic supplements were added to the medium, including 0.8% Wolfe’s vitamins, 100 µM ferric malate, and 570 µM cysteine-HCl. The medium was then incubated overnight to remove oxygen, with cysteine serving as the reducing agent. Subsequently, 1% seed culture was inoculated anaerobically into the medium and incubated at 28°C for 3 days. *F. magnetotacticus* FSS-1 was cultured anaerobically at 28°C for 7 days in a defined medium (all of the sulfate salts were replaced with chloride salts) containing 0.8 g/L of sodium fumarate, as previously described (30). The cultivation procedure followed the same steps as for RS-1. When necessary, antibiotics were added at the following concentrations: for AMB-1, kanamycin at 5 µg/mL; for *E. coli*, kanamycin at 20 µg/mL and ampicillin at 50 µg/mL.

### Large-scale cultivation of RS-1

Large-scale cultivation of RS-1 was conducted in a 10 L glass bottle. RS-1 growth medium (10 L) was added to the bottle and autoclaved. After bubbling the medium with nitrogen gas for 20 min, 0.8% Wolfe’s vitamins solution, 100 µM ferric malate, and 570 µM cysteine-HCl were added anaerobically. The medium was then bubbled again with nitrogen gas for another 20 min and incubated overnight. The color change of resazurin indicated the removal of dissolved oxygen. A 100 mL seed culture was inoculated anaerobically into the medium, which was then cultivated at room temperature in the dark until the early stationary phase. RS-1 cells were harvested by centrifugation at 8,000 × *g* for 10 min at room temperature and stored at −80°C until further use. Approximately 4 g (wet weight) of cells were obtained from the 10 L culture.

### Cellular fractionation

RS-1 cells were suspended in 10 mM Tris-HCl buffer (pH 8.0) and disrupted using a Branson Model 450 ultrasonic oscillator (20 kHz, 80 W) for 10 min. The resulting lysate was centrifuged at 8,000 × *g* for 15 min. The pellet was used for magnetosome isolation, while the supernatant (cell-free extract) was used to separate the membrane and soluble fractions. The pellet was resuspended in 10 mM Tris-HCl buffer (pH 8.0), and magnetosomes were collected magnetically using a neodymium magnet. The magnetosomes were then resuspended in the same buffer and recollected magnetically to wash them. This washing step was repeated five times to obtain the purified magnetosome fraction. The cell-free extract was ultracentrifuged at 100,000 × *g* for 1 h; the pellet was used as the membrane fraction and the supernatant as the soluble fraction. All purification steps were performed at 4°C. Proteins from the soluble, membrane, and purified magnetosome fractions were extracted by treatment with 2% SDS at 37°C overnight. Protein concentrations were determined using the BCA method (Pierce^TM^ BCA Protein Assay Kit, Thermo Scientific).

### Plasmid constructions

The plasmids and primers used in this study are listed in Table S1 and S2, respectively. Detailed procedures for plasmid construction are provided in the Supplemental Text.

### Purification of His-tagged recombinant Mad28

*E. coli* BL21(DE3) containing the pET29b-mad28-His plasmid was grown to the mid-log phase, and expression of C-terminal His-tagged Mad28 was induced with 0.1 mM IPTG for 6 h at 18°C. Cells were harvested and resuspended in lysis buffer (10 mM Tris-HCl, pH 7.5; 25 mM KCl; 10 mM EDTA; 1 mM DTT). Cell disruption was performed using a French press at 1,100 MPa. The lysate was centrifuged at 8,000 × *g* for 10 min. Glycerol (final concentration: 10%) was added to the resulting supernatant to prevent Mad28 aggregation. The mixture was then ultracentrifuged at 100,000 × *g* for 1 h. The resulting pellet (membrane fraction) contained Mad28 (Fig. S1). The membrane fraction was washed in lysis buffer containing 0.5 M NaCl and 10% glycerol for over 1 h to remove peripheral membrane proteins. It was subsequently ultracentrifuged again at 100,000 × *g* for 1 h. The pellet was resuspended in a depolymerization buffer (10 mM Tris-HCl, pH 7.4; 25 mM KCl; 10 mM EDTA; 1 mM DTT; 10% glycerol). To the suspension, 0.5 M NaCl and 1% Triton were added, and the mixture was incubated overnight. The soluble fraction was ultracentrifuged at 100,000 × *g* for 1 h. The supernatant was dialyzed twice for 6 h against lysis buffer (50 mM NaH_2_PO_4_, pH 8.0; 300 mM NaCl; 10 mM imidazole; 1 mM DTT; 10% glycerol). The dialyzed fraction was subjected to Ni-affinity chromatography using Ni-nitrilotriacetic acid agarose (Qiagen), following the Qiagen technical manual. Eluted Mad28 fractions were desalted in buffer A (10 mM Tris-HCl, pH 7.4; 1 mM DTT; 10% glycerol) and concentrated by ultrafiltration. All purifications were conducted using the antigen for generating anti-Mad28 antibodies (Fig. S1A). All purification steps were conducted at 4°C.

### Immunoblotting analysis

Immunoblotting was performed as previously described (45). Proteins separated using SDS-PAGE (Mini-PROTEIN TGX Gels, Bio-Rad) were transferred onto polyvinylidene fluoride membranes (Trans-Blot Turbo Transfer Pack, Bio-Rad) using electroblotting under the Mixed MW protocol, according to the Bio-Rad kit manual. Anti-Mad28 antibody immunoreactivity was detected at a 1:10,000 dilution. Goat anti-rabbit IgG conjugated to horseradish peroxidase (Cytiva) was used at a 1:100,000 dilution in conjunction with Pierce ECL Plus Western blotting detecting reagents (Thermo). Chemifluorescent signals were acquired using a FUSION SOLO S luminescent image analyzer (Vilber). Band intensities were quantified using the Fusion Solo 7S Edge system (Vilber).

### Immunofluorescence analysis

Immunofluorescence staining was performed as previously described (46), with minor modifications. Anti-Mad28 antisera were used at dilutions of 1:250 for RS-1 and 1:500 for FSS-1 cells. The secondary antibody, Alexa Fluor^®^ 488 (Molecular Probes), was applied at a 1:1,000 dilution.

### Correlative light and electron microscopy

RS-1 cells were collected by centrifugation and fixed with 2% paraformaldehyde. Formvar- and carbon-coated grids were coated with 0.005% poly-L-lysine. The coated grids were placed on a drop containing RS-1 cells for 10 min and then air-dried. The grids were stored at −20°C until further use. Cells on the grids were washed twice with a drop of PBS buffer for 5 min. They were then treated with lysozyme buffer (50 mM Tris-HCl, pH 8.0; 500 µg/ml EDTA; 100 µg/ml lysozyme; 0.5 M sucrose; 1% Triton X-100) for 30 s. Immediately afterward, the permeabilized cells were washed twice with a drop of PBS buffer for 5 min. The grids were incubated on a drop of PBS buffer containing 1% BSA for 10 min in a moist chamber. The cells were then incubated on a drop of primary anti-Mad28 antibody solution, diluted 1:250 in PBS buffer supplemented with 0.5% BSA, for 30 min. The grids were washed three times with drops of PBS buffer containing 0.5% BSA for 5 min each. Next, the grids were incubated for 30 min on a drop of secondary antibody solution (Alexa Fluor^®^ 488, Molecular Probes), diluted 1:1,000 in PBS buffer supplemented with 0.5% BSA. The grids were washed again three times with drops of PBS buffer containing 0.5% BSA for 5 min each, followed by two washes with drops of distilled water for 2 min each. The grids were then air-dried. Mad28 localization in cells was visualized using a total internal reflection fluorescence (TIRF) microscopy-based system on an inverted microscope (Nikon Ti-E) equipped with a 100 × CFI Apo TIRF objective lens and a 1.5 × C-mount adapter (Nikon). A 488 nm laser (Sapphire; Coherent) was used to illuminate the sample at an inclined angle, slightly steeper than the critical angle required for total internal reflection, allowing illumination of the entire bacterial cell. The laser beam angle was manually adjusted to optimize the signal-to-noise ratio. Finally, the cells on the grids were examined using a JEOL JEM-2100Plus TEM.

### HIRO microscopy

Bacteria were imaged as previously described (21) using a TIRF microscopy-based system on an inverted microscope (Nikon Ti-E) equipped with a 100 × CFI Apo TIRF objective lens and a 1.5 × C-mount adapter (Nikon). Exposure times ranged from 0.1 to 1 s for Dendra2 and 50 to 100 ms for bright-field images, with images acquired at 1-minute intervals. Illumination was applied only during image acquisition.

### Observation of Mad28 expression in BL21(DE3)

Mad28-Dendra2 expression was induced from the pBBR111-mad28-Dendra2 plasmid in *E. coli* BL21(DE3). Cells (3 mL) were grown to the mid-log phase in LB medium, and isopropyl β-D-1-thiogalactopyranoside (IPTG) was added to a final concentration of 1 mM to induce Mad28-Dendra2 fusion protein. The culture was incubated at 30°C for 5 h.

### Generation of curved-shaped *E. coli*

The pET15b-CreS vector and/or pBBR111-mad28-Dendra2 plasmid were introduced into *E. coli* HMS174. Cells (3 mL) were grown to the mid-log phase in M9 medium supplemented with 0.2% glycerol, as previously described (38). Cephalexin was added to a final concentration of 40 µM/mL to inhibit cell division, and IPTG was added to a final concentration of 0.1 mM to induce the expression of Mad28-Dendra2 fusion proteins. The culture was incubated overnight at 30°C.

### Liposome pelleting assay

Liposomes were prepared using *E. coli* lipid extract (Avanti Polar Lipid, USA), as previously described (47). The resulting liposomes were resuspended in Polymerization buffer (10 mM Tris-HCl, pH 7.5; 25 mM KCl; 1 mM MgCl_2_; 1 mM DTT). Liposomes (0.66 mg/mL) were incubated with 1 µM each of Mad28, MamK, or BSA for 1 h. The mixtures were then ultracentrifuged at 100,000 × *g* for 1 h. Pellets and supernatants were analyzed using SDS-PAGE. Control experiments were performed using Polymerization buffer alone or Polymerization buffer supplemented with 1% Triton X-100 to solubilize the liposomes.

## SUPPLEMENTAL MATERIAL

Supplemental figures jb.00368-25-s0001.pdf Figure S1 to S10.

Supplemental materials jb.00368-25-s0002.doc Supplemental material and methods, Fig. S1 to S10 legends, Movie S1 to S4 legends, Tables S1 and S2, and References.

Movie S1 jb.00368-25-s0003.avi Magnetosome motion in ΔmamK AMB-1 cells expressing MamKRS-1.

Movie S2 jb.00368-25-s0004.avi Magnetosome motion in ΔmamK AMB-1 cells expressing Mad28RS-1.

Movie S3 jb.00368-25-s0005.avi Low-magnification images of magnetosome motion in ΔmamK AMB-1 cells expressing MamKRS-1.

Movie S4 jb.00368-25-s0006.avi Low-magnification images of magnetosome motion in ΔmamK AMB-1 cells expressing Mad28RS-1.

ASM does not own the copyrights to Supplemental Material that may be linked to, or accessed through, an article. The authors have granted ASM a non-exclusive, world-wide license to publish the Supplemental Material files. Please contact the corresponding author directly for reuse.
