# Supplementary material for: Mad28, a conserved actin-like protein in deep-branching magnetotactic bacteria, exhibits cell curvature-dependent localization
Source: J Bacteriol. 2025 Nov 24;207(12):e00368-25. doi: 10.1128/jb.00368-25 (PMC12713370; doi:10.1128/jb.00368-25)
Supplement: Supplemental figures — Figure S1 to S10. [file jb.00368-25-s0001.pdf]

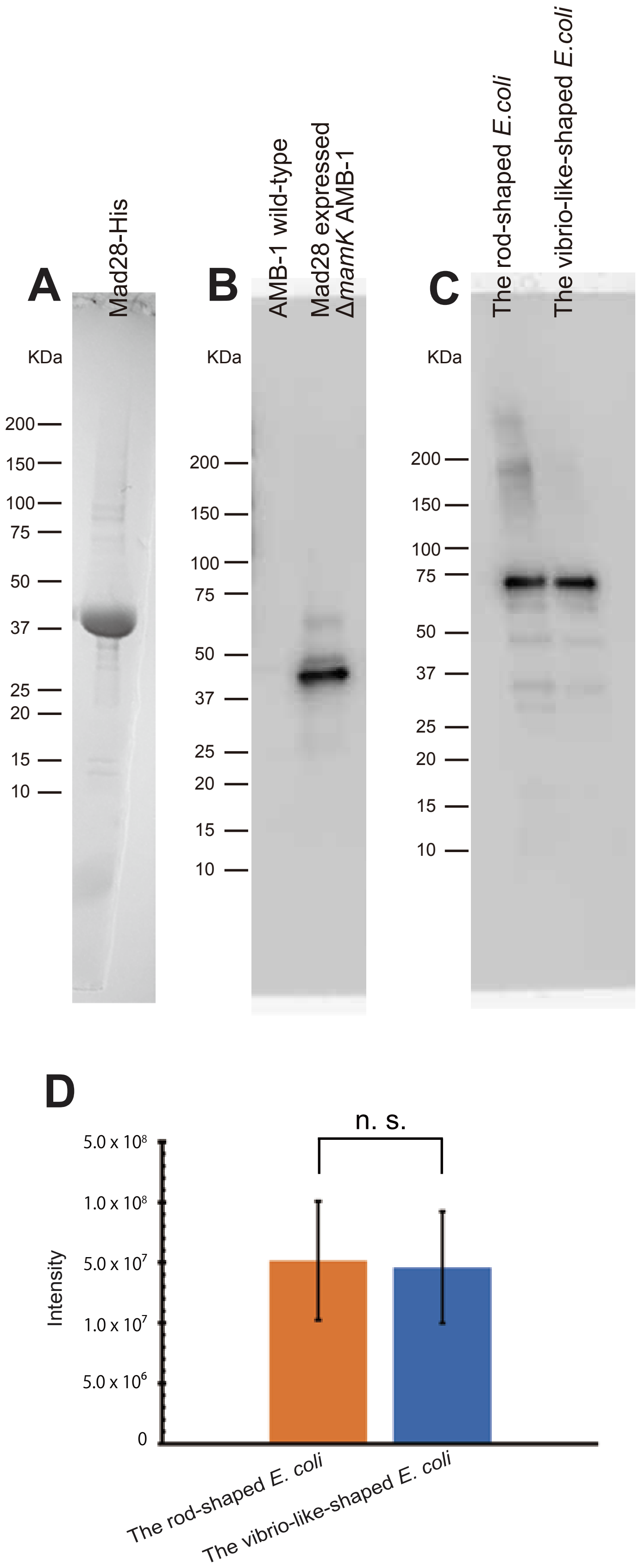

FIG S1 Purified Mad28-His and immunoblotting of Mad28 expressed Δ*mamK* AMB-1, the rod- and vibrio-like-shaped *E. coli*. (A) SDS-PAGE profile stained with Coomassie Brilliant Blue, showing the purified C-terminal His-tagged Mad28 from *E. coli* BL21(DE3). (B) Immunoblotting with anti-Mad28^{RS-1} antibody of lysates from AMB-1 wild-type and Δ*mamK* AMB-1 expressing Mad28. Proteins (7.5 µg per lane) extracted from both samples were loaded on each lane. Mad28 is indicated by the most intense bands with apparent molecular masses of approximately 42 kDa. (C) Immunoblotting with anti-Mad28^{RS-1} antibody from lysates expressing Mad28-Dendra2 in either wild-type rod-shaped *E. coli* cells or vibrio-like-shaped *E. coli* cells expressing CreS. Proteins (10 µg per lane) extracted from each lysate were loaded on each lane. Mad28-Dendra2 is indicated by the most intense bands with apparent molecular masses of approximately 70 kDa. (D) Quantification of the relative expression levels of Mad28 in rod-shaped and vibrio-like-shaped *E. coli*, based on the band intensities from the immunoblots (n = 5).

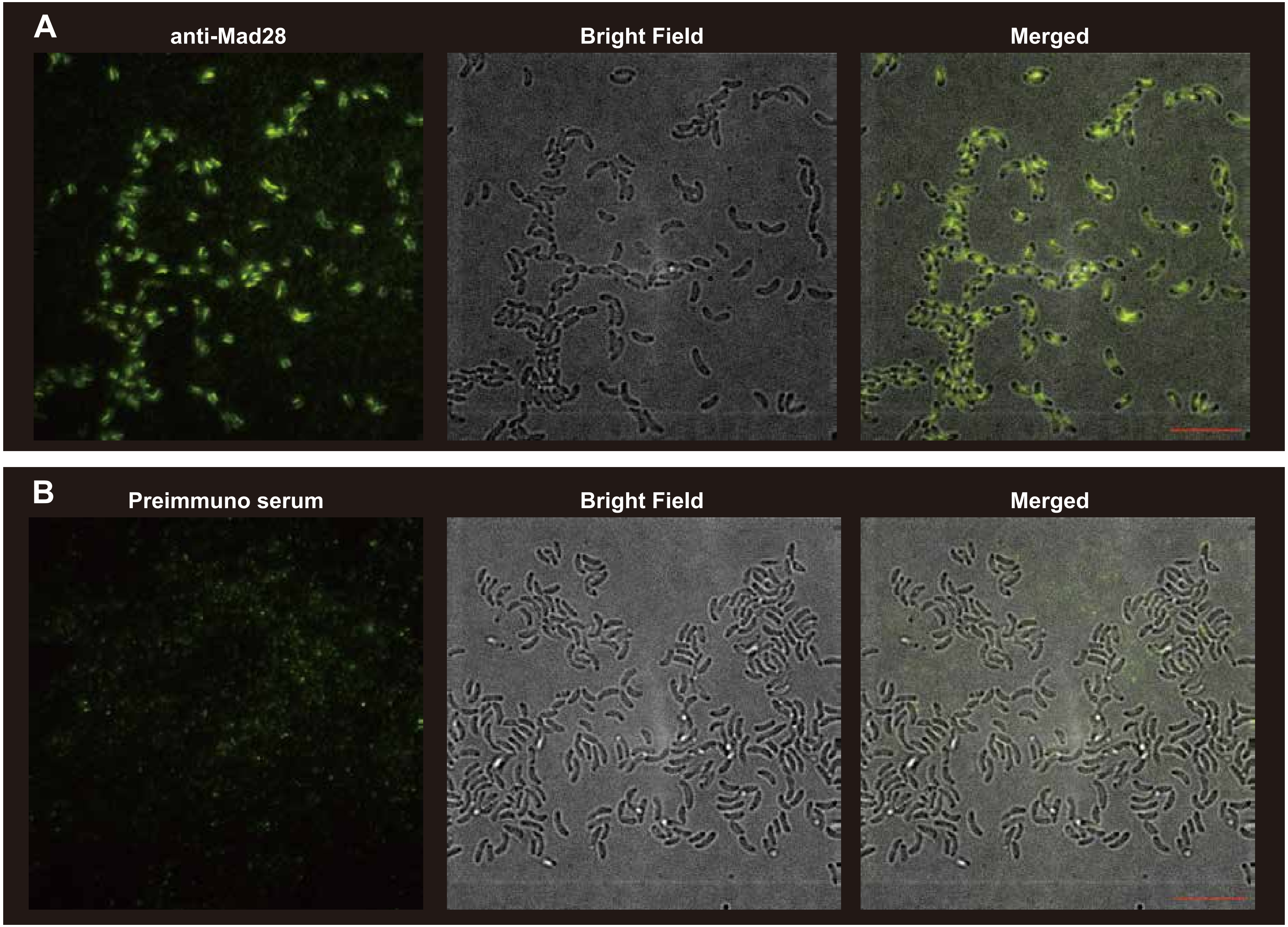

FIG S2 Low-magnification images of immunofluorescence-stained RS-1 cells. Immunofluorescence staining of RS-1 cells using anti-Mad28 antibodies (A) and preimmune serum (B) as primary antibody. Fluorescence microscopy images (Alexa Fluor® 488), bright-field images, and merged images are shown. All images were acquired under similar conditions and processed uniformly for contrast adjustment, as described in the Materials and Methods section. Scale bars: 10 µm.

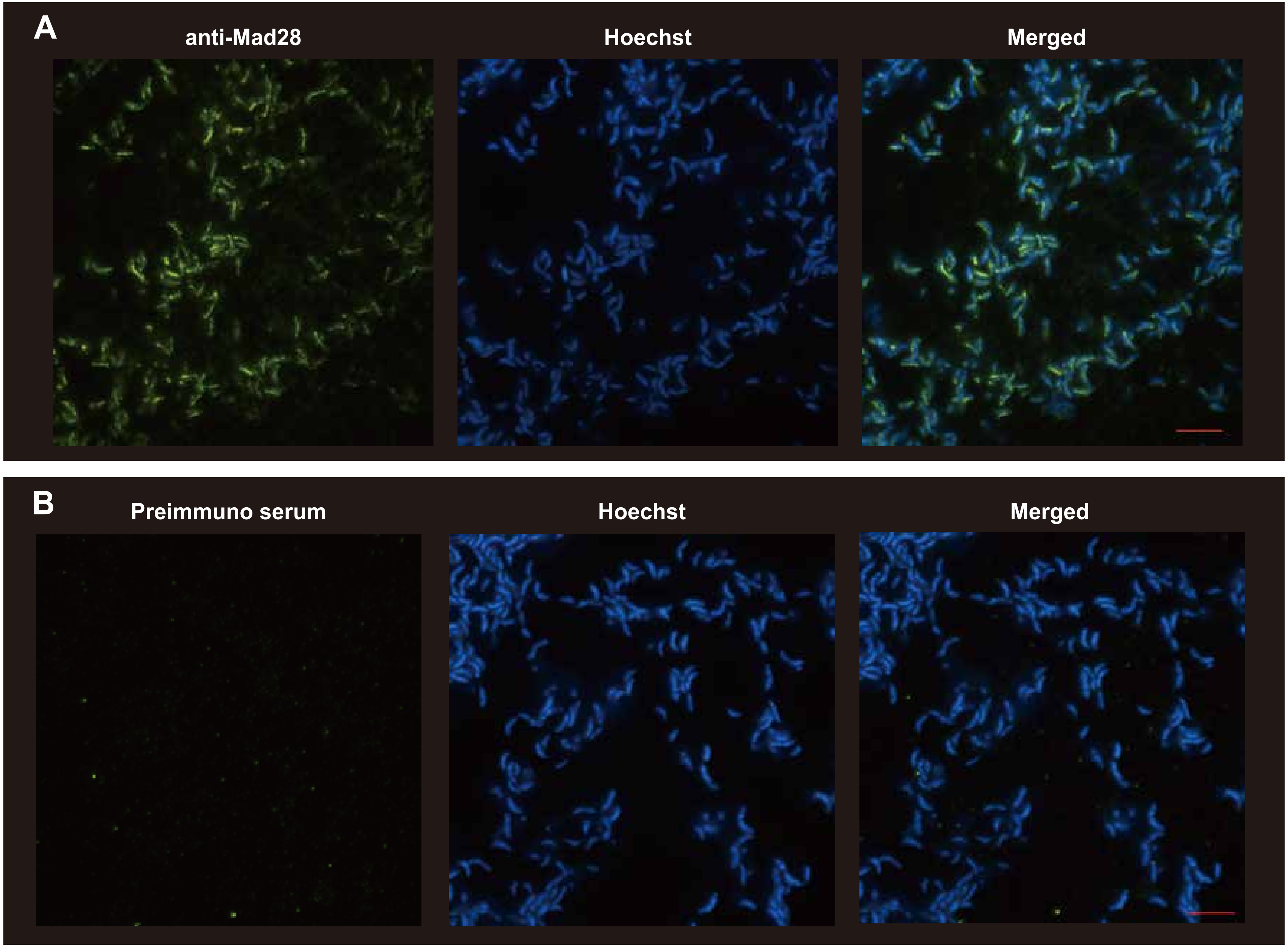

FIG S3 Low-magnification images of immunofluorescence-stained FSS-1 cells. Immunofluorescence staining of FSS-1 cells using anti-Mad28 antibodies (A) and preimmune serum (B) as primary antibody. Fluorescence microscopy images (Alexa Fluor® 488), bright-field images, and merged images are shown. All images were acquired under similar conditions and processed uniformly for contrast adjustment, as described in the Materials and Methods section. Scale bars: 10 µm.

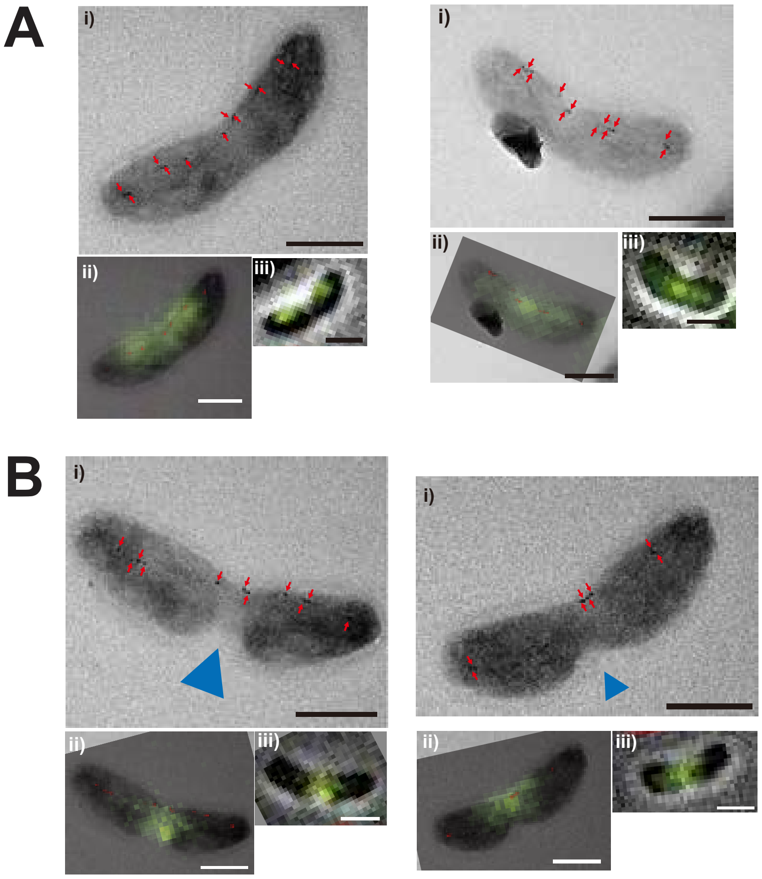

FIG S4 CLEM images of RS-1 cells showing Mad28 localization Pattern I (A) and Pattern II (B). (i) TEM images of RS-1 cells. Red arrows indicate magnetite crystals, and the blue arrowhead denotes the constriction sites. (ii) CLEM images, with magnetite crystals shown in red. (iii) Immunofluorescence images superimposed on bright-field images. Scale bars: 1 µm.

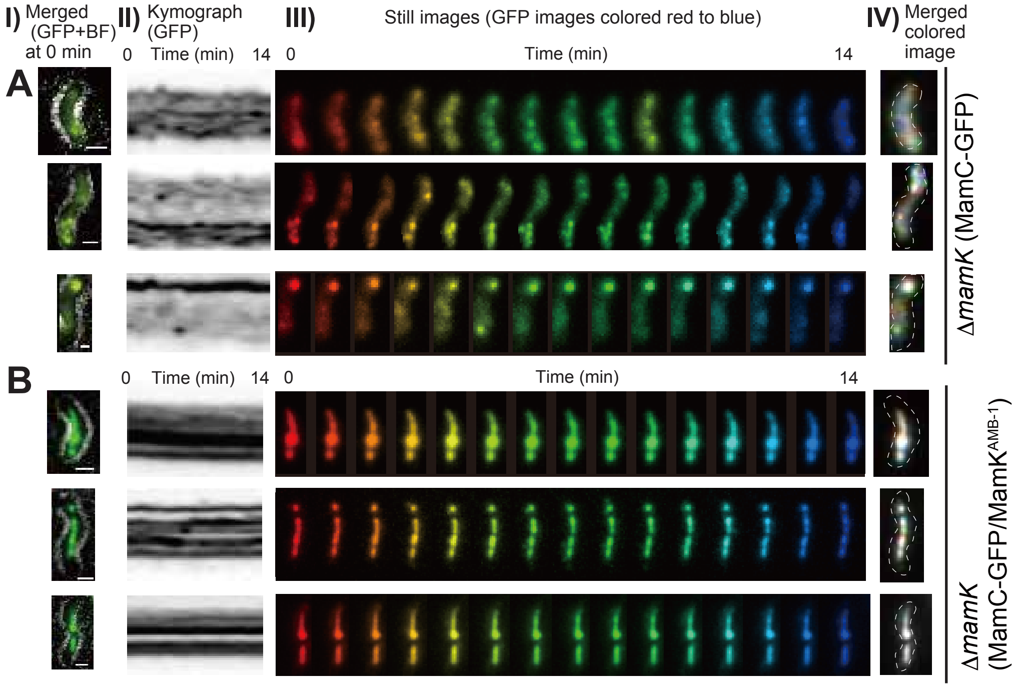

FIG S5 Rescue of MamK-dependent static magnetosome positioning. Magnetosomes were visualized using MamC-GFP in (A) uncomplemented Δ*mamK* AMB-1 cells and (B) $MamK^{AMB-1}$. (Column I): Merged GFP and bright-field images of cells at time zero. Scale bars: 1 µm. (Column II): Kymographs showing GFP signal trajectories in maximum projections. (Column III): Time-lapse still images acquired over a 14-min interval, sequentially rainbow-colored, red to blue. (Column IV): Merged images of the rainbow-colored still images in Column III. White signals indicate static GFP fluorescence, while colored signals represent dynamic GFP fluorescence. The images show that magnetosomes remained static in $MamK^{AMB-1}$ but dynamic in uncomplemented Δ*mamK* cells.

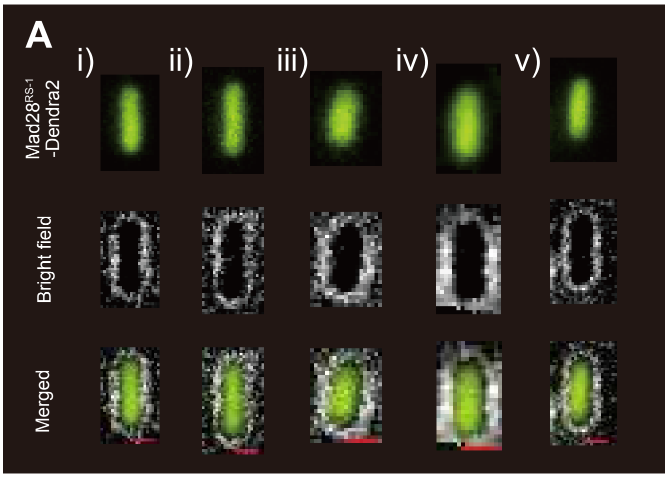

FIG S6 Heterologous expression of Dendra2-Mad28$^{RS\text{-}1}$ in *E. coli*. Scale bars: 1 μm.

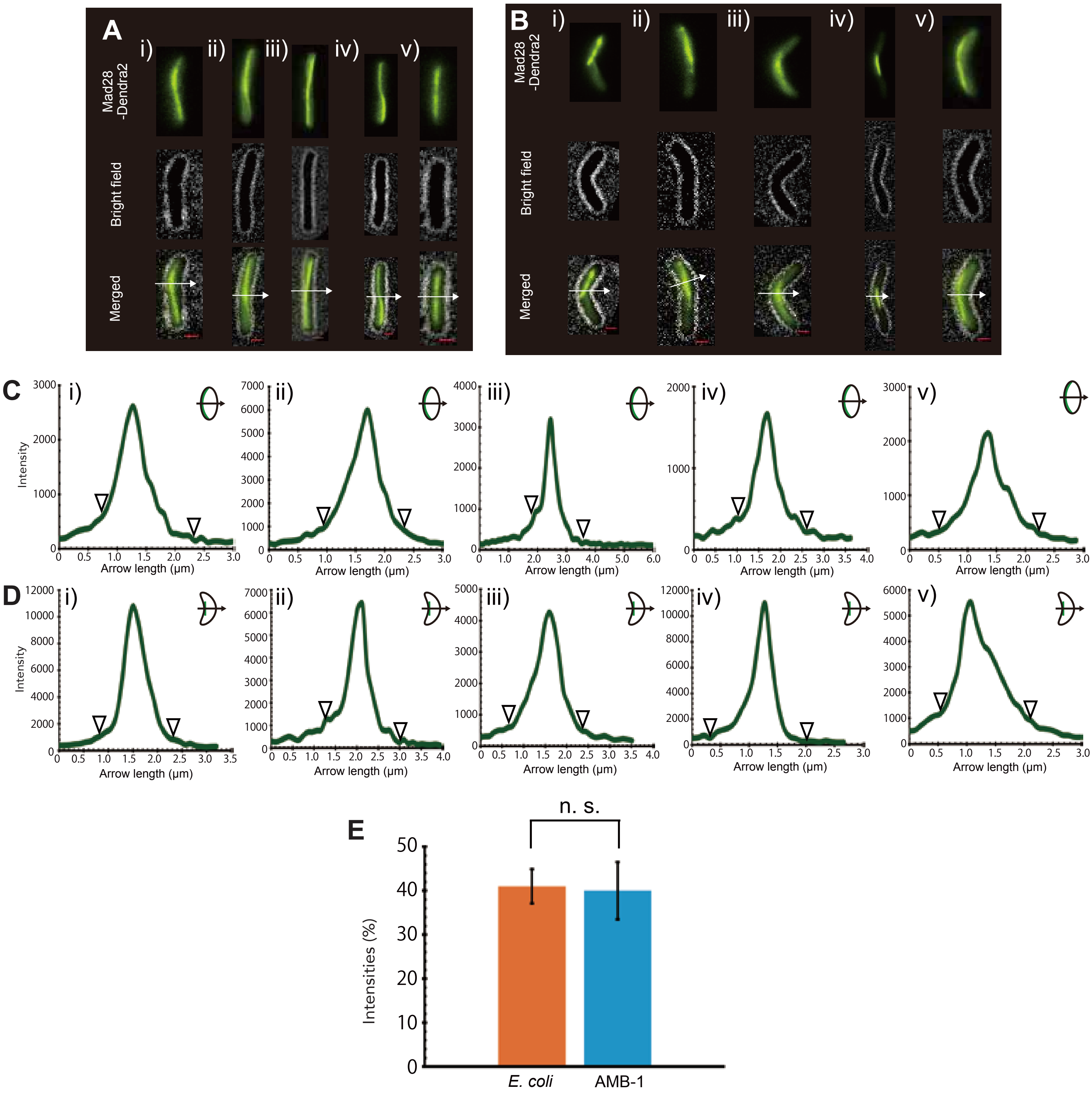

FIG S7 Heterologous expression of Mad28[RS-1] in rod-shaped (A) and vibrio-like-shaped (B) *E. coli* cells. White arrows indicate the direction and position of fluorescence intensity measurements. Scale bar: 1 μm. (C, D) Transverse fluorescence intensity profiles of Mad28[RS-1]-Dendra2 in rod-shaped (C) and vibrio-like-shaped (D) *E. coli* cells along the white arrows. White arrowheads indicate the edge of cells. (E) Percentage intensities of three pixels corresponding to the peak tips in the Mad28 localization line profiles (n = 50).

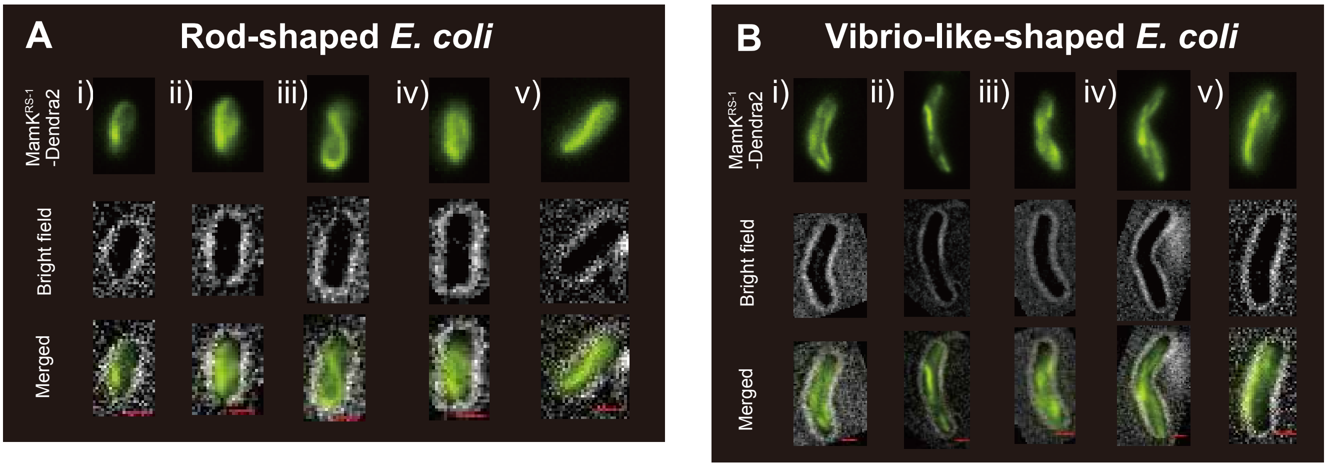

FIG S8 Heterologous expression of MamK^{RS-1} in *E. coli*. MamK^{RS-1}-Dendra2 expression in rod-shaped (A) and vibrio-like (B) *E. coli* cells. Scale bars: 1 μm.

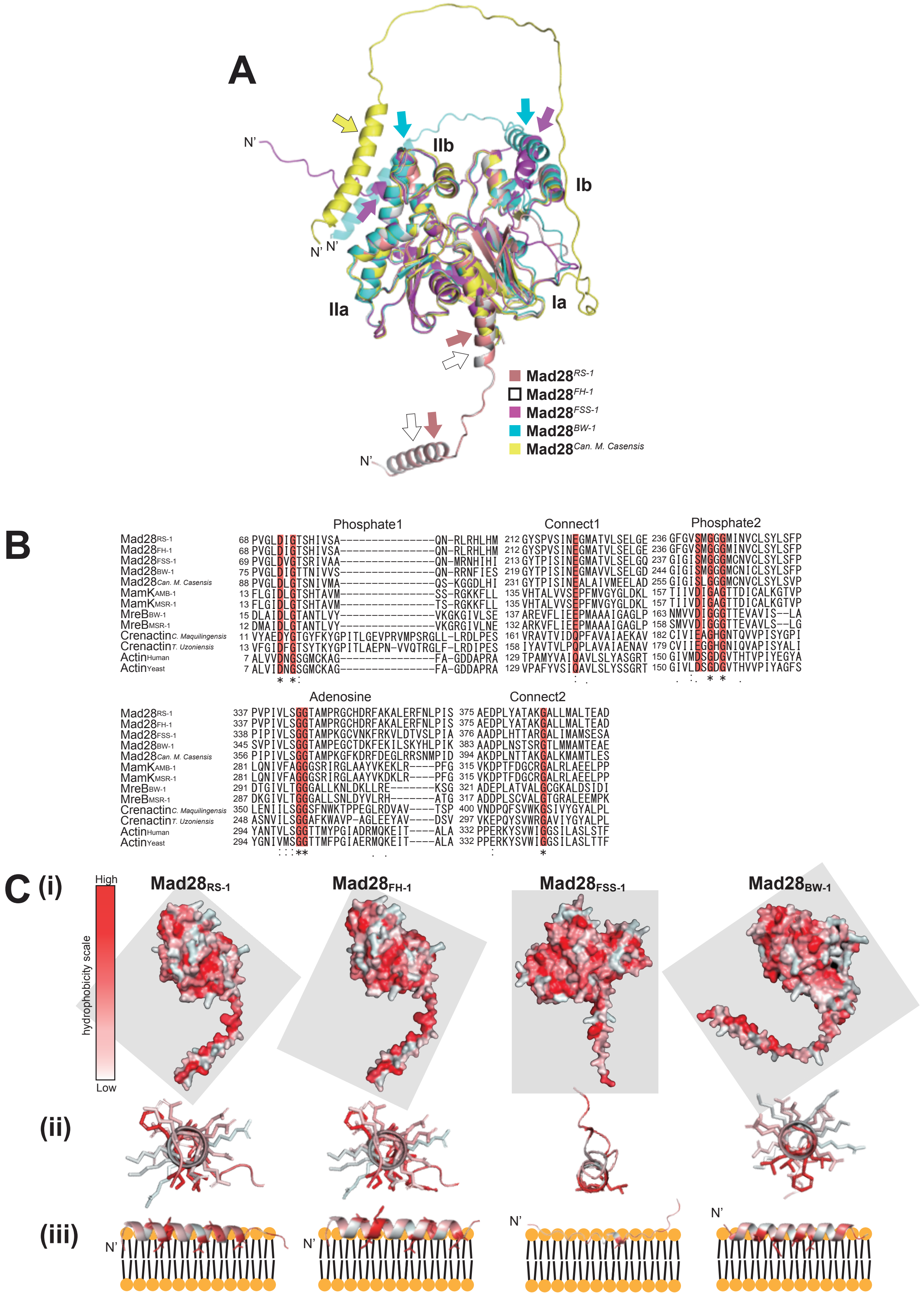

FIG S9 Predicted three-dimensional structure of Mad28 analyzed using AlphaFold2. (A) Alignment of three-dimensional (3D) structure models of Mad28 generated using PyMOL. The structure retains four conserved subdomains of actin-like structure and an uncharacterized N-terminal region. Colored arrows indicate α-helices. (B) Multiple alignment of representative actin-like proteins and known Mad28 sequences for the five sequence motifs. Amino acids colored by red indicate ATP binding sites and a putative interdomain hinge. (C) Predicted 3D structure of individual Mad28 using AlphaFold2 and visualized in PyMOL. (i) Amphipathic structure of Mad28, with amino acids colored according to hydrophobicity: hydrophilic residues in white and hydrophobic residues in red. (ii) Localization of hydrophobic amino acids within the amphipathic α-helix at the N-terminus. (iii) Model of the N-terminal amphipathic α-helix of Mad28 embedded on the lipid bilayer.

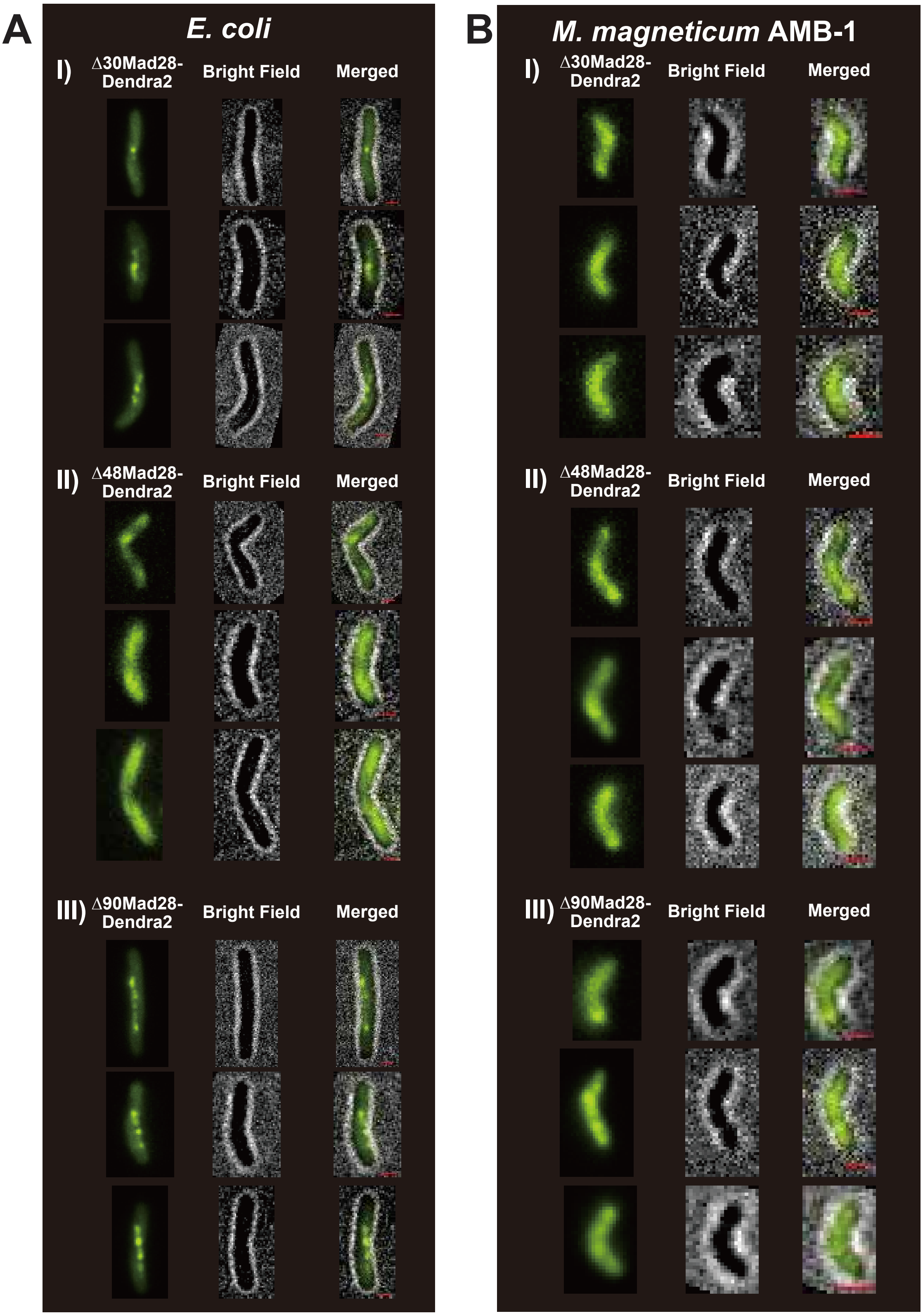

FIG S10 Heterologous expression of truncated Mad28^{RS-1} in vibrio-like-shaped *E. coli* (A) and AMB-1 cells. (Ai, ii, iii) Truncated Mad28 variants lacking the first 30, 48, and 90 amino acids, respectively, were coexpressed with CreS in *E. coli*. (Bi, ii, iii) The same Mad28 truncations (Δ1–30, 1–48, 1–90) were expressed in AMB-1 wild-type cells. Scale bars: 1 μm.
