# Supplementary material for: Mad28, a conserved actin-like protein in deep-branching magnetotactic bacteria, exhibits cell curvature-dependent localization
Source: J Bacteriol. 2025 Nov 24;207(12):e00368-25. doi: 10.1128/jb.00368-25 (PMC12713370; doi:10.1128/jb.00368-25)
Supplement: Supplemental materials — Supplemental material and methods, Fig. S1 to S10 legends, Movie S1 to S4 legends, Tables S1 and S2, and References. [file jb.00368-25-s0002.doc]

Supplementary materials

**Mad28, a Conserved Actin-like Protein in Deep-Branching Magnetotactic Bacteria, Exhibits Cell Curvature-Dependent Localization**

Rino Shimoshige,^a^, Hirokazu Shimoshige,^b,c^, Azuma Taoka,^d,e^#

^a^Graduate School of Natural Science and Technology, Kanazawa University

^b^Bio-Nano Electronics Research Centre, Toyo University

^c^Institute for Extra-cutting-edge Science and Technology Avant-garde Research (X-star), Japan Agency for Marine-Earth Science and Technology (JAMSTEC)

^d^Institute of Science and Engineering, Kanazawa University

^e^Nano Life Science Institute (WPI-NanoLSI), Kanazawa University,

Contents

- Supplemental text

Supplemental material and methods

- Supplementary figure legends
- Supplementary movie legends
- Supplementary tables S1
- Supplementary tables S2
- References

**Supplementary material**

**Supplementary Materials and Methods**

**Plasmid constructions**

The plasmids and primers used in this study are listed in Tables S1 and S2, respectively. To express recombinant proteins in *E. coli* cells, pET29b (Novagen), pET15b (Novagen), and the broad-host-range protein expression vector pBBR111—harboring the isopropyl-D-thiogalactopyranoside-inducible *tac* promoter (1)—were used. pBBR111 was also used for expression in AMB-1 cells. Cloning procedures were performed using an infusion cloning system (In-Fusion Snap Assembly Master Mix, TaKaRa).

**pET29b-mad28-His:** This plasmid was constructed to express Mad28 for protein purification as an antigen. The DNA fragment encoding *mad28* was PCR-amplified using the template pEX-A2J2-Mad28_RS-1 and the primers “mad28_RS-1_f” and “His-insert-R.” The amplicons were inserted into the *Nde*I and *EcoR*I sites of pET29b. For infusion cloning, pET29b was linearized using PCR with the primers “His-linear-F” and “pET29_r.”

**pBBR111-mamC-GFP/mad28^RS-1^:** This plasmid was constructed to coexpress MamC-GFP and Mad28 for complementation experiments in Δ*mamK* AMB-1 cells. The DNA fragment encoding *mad28* was PCR-amplified using the template pET29b-mad28-His and the primers “Mad28f1” and “Mad28r1.” The amplicons were inserted into the MamK^AMB-1^ site of pBBR_mamC-gfp/mamK^AMB-1^. For infusion cloning, pBBR_mamC-gfp/mamK^AMB-1^ (2) was linearized using PCR with the primers “pBBR_CG_f1” and “pBBR_CG_r1.” The resulting plasmids were introduced into AMB-1 cells through conjugation with *E. coli* WM3064, as previously described (3).

**pBBR111-mamC-GFP/mamK^RS-1^:** This plasmid was constructed to coexpress MamC-GFP and MamK from RS-1 for complementation experiments in Δ*mamK* AMB-1 cells. The DNA fragment encoding *mamK* was PCR-amplified using the template pEX-A2J2-MamK_RS-1 and the primers “MamKRS1_f1” and “MamKRS1_r1.” The amplicons were inserted into the MamK^AMB-1^ site of pBBR_mamC-gfp/mamK^AMB-1^. For infusion cloning, pBBR_mamC-gfp/mamK^AMB-1^ (2) was linearized using PCR with the primers “pBBR_CG_f1” and “pBBR_CG_r1.” The resulting plasmids were introduced into AMB-1 cells through conjugation with *E. coli* WM3064, as previously described (3).

**pET15b-creS:** This plasmid was constructed to induce cell curvature in *E. coli*. The DNA fragment encoding *creS* was PCR-amplified using the template pEX-A2J2_cresentin and the primers “creS forward primer” and “creS reverse primer.” The amplicons were inserted into the *Nla*III and *Bam*HI sites of the pET15b vector. For infusion cloning, pET15b was linearized using PCR with the primers “pET15b forward primer” and “pET15b reverse primer.”

**pBBR111-mad28-Dendra2:** This plasmid was constructed to visualize Mad28 localization in *E. coli* and AMB-1 cells. The first DNA fragment encoding *mad28* was PCR-amplified using the template pET29b-mad28-His, and the primers “C terminal 28_F” and “C terminal 28_R.” The second DNA fragment, encoding a linker sequence for ligation downstream of *mad28,* was PCR-amplified using the template pEX-kondo_linker and the primers “N terminal linker forward” and “N terminal linker reverse.” The third DNA fragment encoding *dendra2* was PCR-amplified using the template pDebdra2 (Clontech) and the primers “N terminal Dd2 forward” and “28Dd2_r.” The amplicons were inserted into the *Xho*I and *Eco*RI sites of pBBR111. For infusion cloning, pBBR111 was linearized using PCR with the primers “N terminal reverse” and “pBBRlinear_f.”

**pBBR111-mamK^RS-1^-Dendra2:** This plasmid was constructed to visualize MamK^RS-1^ localization in *E. coli* cells. The first DNA fragment encoding *mamK^RS-1^* was PCR-amplified using the template pEX-A2J2-MamK_RS-1, and the primers “K_RS1_Den_f2” and “K_RS1_Den_r2.” The second DNA fragment, encoding a linker sequence for ligation downstream of *mamK,* was PCR-amplified using the template pEX-kondo_linker and the primers “N terminal linker forward” and “N terminal linker reverse.” The third DNA fragment encoding *dendra2* was PCR-amplified using the template pDebdra2 (Clontech) and the primers “N terminal Dd2 forward” and “28Dd2_r.” The amplicons were inserted into the *Xho*I and *Eco*RI sites of pBBR111. For infusion cloning, pBBR111 was linearized using PCR with the primers “N terminal reverse” and “pBBRlinear_f.”

**pBBR111-Dendra2-mad28:** This plasmid was constructed to visualize Mad28 localization in *E. coli* cells. The DNA fragment encoding *mad28* was PCR-amplified using the template pET29b-mad28-His and the primers “N terminal 28_F” and “N terminal 28_R.” The second DNA fragment, encoding a linker sequence for ligation upstream of *mad28,* was PCR-amplified using the template pEX-kondo_linker and the primers “N terminal linker forward” and “N terminal linker reverse.” The third DNA fragment encoding *dendra2* was PCR-amplified using the template pDebdra2 (Clontech) and the primers “C terminal Dd2 forward” and “C terminal Dd2 reverse.” The amplicons were inserted into the *Xho*I and *Eco*RI sites of pBBR111. For infusion cloning, pBBR111 was linearized using PCR with the primers “N terminal reverse” and “Den-K_RS-1_f1.”

**pBBR111-Dendra2*-*mamK^RS-1^:** This plasmid was constructed to visualize MamK localization in *E. coli* cells. The DNA fragment encoding *mamK^RS-1^* was PCR-amplified using the template pEX-A2J2-MamK_RS-1 and the primers “Den-K_RS-1_f2” and “Den-K_RS-1_r2.” The second DNA fragment, encoding a linker sequence ligation upstream of *mamK^RS-1^,* was PCR-amplified using the template pEX-kondo_linker and the primers “N terminal linker forward” and “N terminal linker reverse.” The third DNA fragment encoding *dendra2* was PCR-amplified using the template pDebdra2 (Clontech) and the primers “C terminal Dd2 forward” and “C terminal Dd2 reverse.” The amplicons were inserted into the *Xho*I and *Eco*RI sites of pBBR111. For infusion cloning, pBBR111 was linearized using PCR with the primers “N terminal reverse” and “Den-K_RS-1_f1.”

**pBBR111-∆30mad28-Dendra2, pBBR111-∆48mad28-Dendra2, and pBBR111-∆90mad28-Dendra2:** These plasmids were constructed to visualize the localization of truncated Mad28 variants in *E. coli* and AMB-1 cells. The pBBR111-mad28-Dendra2 plasmid was linearized using PCR with the phosphorylated primer “N terminal reverse” in combination with “delta1-30-F,” “delta1-48-F,” or “delta1-90-F,” respectively. The purified PCR product was ligated using the DNA Ligation Kit, following the manufacturer’s protocol (Takara).

**Supplementary Figure Legends**

**Fig. S1.** Purified Mad28-His and immunoblotting of Mad28 expressed Δ*mamK* AMB-1, the rod- and vibrio-like-shaped *E. coli*. (A) SDS-PAGE profile stained with Coomassie Brilliant Blue, showing the purified C-terminal His-tagged Mad28 from *E. coli* BL21(DE3). (B) Immunoblotting with anti-Mad28^RS-1^ antibody of lysates from AMB-1 wild-type and Δ*mamK* AMB-1 expressing Mad28. Proteins (7.5 µg per lane) extracted from both samples were loaded on each lane. Mad28 is indicated by the most intense bands with apparent molecular masses of approximately 42 kDa. (C) Immunoblotting with anti-Mad28^RS-1^ antibody from lysates expressing Mad28-Dendra2 in either wild-type rod-shaped *E. coli* cells or vibrio-like-shaped *E. coli* cells expressing CreS. Proteins (10 µg per lane) extracted from each lysate were loaded on each lane. Mad28-Dendra2 is indicated by the most intense bands with apparent molecular masses of approximately 70 kDa. (D) Quantification of the relative expression levels of Mad28 in rod-shaped and vibrio-like-shaped *E. coli,* based on the band intensities from the immunoblots (n = 5).

**Fig. S2.** Low-magnification images of immunofluorescence-stained RS-1 cells. Immunofluorescence staining of RS-1 cells using anti-Mad28 antibodies (A) and preimmune serum (B) as primary antibody. Fluorescence microscopy images (Alexa Fluor® 488), bright-field images, and merged images are shown. All images were acquired under similar conditions and processed uniformly for contrast adjustment, as described in the Materials and Methods section. Scale bars: 10 μm.

**Fig. S3.** Low-magnification images of immunofluorescence-stained FSS-1 cells. Immunofluorescence staining of FSS-1 cells using anti-Mad28 antibodies (A) and preimmune serum (B) as primary antibody. Fluorescence microscopy images (Alexa Fluor® 488), bright-field images, and merged images are shown. All images were acquired under similar conditions and processed uniformly for contrast adjustment, as described in the Materials and Methods section. Scale bars: 10 μm.

**Fig. S4.** CLEM images of RS-1 cells showing Mad28 localization Pattern I (A) and Pattern II (B). (i) TEM images of RS-1 cells. Red arrows indicate magnetite crystals, and the blue arrowhead denotes the constriction sites. (ii) CLEM images, with magnetite crystals shown in red. (iii) Immunofluorescence images superimposed on bright-field images. Scale bars: 1 μm.

**Fig. S5.** Rescue of MamK-dependent static magnetosome positioning. Magnetosomes were visualized using MamC-GFP in (A) uncomplemented Δ*mamK* AMB-1 cells and (B) MamK^AMB-1^. (Column I): Merged GFP and bright-field images of cells at time zero. Scale bars: 1 μm. (Column II): Kymographs showing GFP signal trajectories in maximum projections. (Column III): Time-lapse still images acquired over a 14-min interval, sequentially rainbow-colored, red to blue. (Column IV): Merged images of the rainbow-colored still images in Column III. White signals indicate static GFP fluorescence, while colored signals represent dynamic GFP fluorescence. The images show that magnetosomes remained static in MamK^AMB-1^ but dynamic in uncomplemented Δ*mamK* cells.

**Fig. S6.** Heterologous expression of Dendra2-Mad28^RS-1^ in *E. coli.* Scale bars: 1 μm.

**Fig. S7.** Heterologous expression of Mad28^RS-1^ in rod-shaped (A) and vibrio-like-shaped (B) *E. coli* cells. White arrows indicate the direction and position of fluorescence intensity measurements. Scale bar: 1 μm. (C, D) Transverse fluorescence intensity profiles of Mad28^RS-1^-Dendra2 in rod-shaped (C) and vibrio-like-shaped (D) *E. coli* cells along the white arrows. White arrowheads indicate the edge of cells. (E) Percentage intensities of three pixels corresponding to the peak tips in the Mad28 localization line profiles (n = 50).

**Fig. S8.** Heterologous expression of MamK^RS-1^ in *E. coli*. MamK^RS-1^-Dendra2 expression in rod-shaped (A) and vibrio-like (B) *E. coli* cells. Scale bars: 1 μm.

**Fig. S9**. Predicted three-dimensional structure of Mad28 analyzed using AlphaFold2. (A) Alignment of three-dimensional (3D) structure models of Mad28 generated using PyMOL. The structure retains four conserved subdomains of actin-like structure and an uncharacterized N-terminal region. Colored arrows indicate α-helices. (B) Multiple alignment of representative actin-like proteins and known Mad28 sequences for the five sequence motifs. Amino acids colored by red indicate ATP binding sites and a putative interdomain hinge. (C) Predicted 3D structure of individual Mad28 using AlphaFold2 and visualized in PyMOL. (i) Amphipathic structure of Mad28, with amino acids colored according to hydrophobicity: hydrophilic residues in white and hydrophobic residues in red. (ii) Localization of hydrophobic amino acids within the amphipathic α-helix at the N-terminus. (iii) Model of the N-terminal amphipathic α-helix of Mad28 embedded on the lipid bilayer.

**Fig. S10**. Heterologous expression of truncated Mad28^RS-1^ in vibrio-like-shaped *E. coli* (A) and AMB-1 cells. (Ai, ii, iii) Truncated Mad28 variants lacking the first 30, 48, and 90 amino acids, respectively, were coexpressed with CreS in *E. coli*. (Bi, ii, iii) The same Mad28 truncations (Δ1–30, 1–48, 1–90) were expressed in AMB-1 wild-type cells. Scale bars: 1 μm.

**Movie S1.** Magnetosome motion in Δ*mamK* AMB-1 cells expressing MamK^RS-1^

**Movie S2.** Magnetosome motion in Δ*mamK* AMB-1 cells expressing Mad28^RS-1^

**Movie S3.** Low-magnification images of magnetosome motion in Δ*mamK* AMB-1 cells expressing MamK^RS-1^

**Movie S4.** Low-magnification images of magnetosome motion in Δ*mamK* AMB-1 cells expressing Mad28^RS-1^

Table S1. The plasmids used in this study.

| **Plasmid** | **Discription** | **Reference** |
| --- | --- | --- |
| pET29b | Protein expression vector for *E. coli* | Novagen |
| pET15b | Protein expression vector for *E. coli* | Novagen |
| pBBR111 | Protein expression vector for AMB-1 and *E. coli* | (1) |
| pDendra2 | The source for *dendra2* gene | Clontech |
| pEX-A2J2-Mad28_RS-1 | The source for the *mad28* gene from RS-1 | This study |
| pEX-A2J2-MamK_RS-1 | The source for the *mamK* gene from RS-1 | This study |
| pEX-A2J2_cresentin | The source for the *creS* gene from *Caulobacter crescentus*. | This study |
| pEX-kondo_linker | The source for the linker gene | This study |
| pET29b-mad28-His | Expression of Mad28-His in *E. coli* | This study |
| pBBR111-mamC-gfp/mad28 | Coexpression for MamC-GFP and Mad28 in AMB-1. | This study |
| pBBR111-mamC-gfp/mamK^RS-1^ | Coexpression for MamC-GFP and MamK^RS-1^ in AMB-1. | This study |
| pBBR111-mamC-gfp | Expression for MamC-GFP in AMB-1. | (2) |
| pBBR111-mamC-gfp/mamK^AMB-1^ | Coexpression for MamC-GFP and MamK^AMB-1^ in AMB-1. | (2) |
| pET15b-crescentin | Expression of CreS in *E. coli* | This study |
| pBBR111-mad28-Dendra2 | Expression of Mad28-Dendra2 in *E. coli* and AMB-1. | This study |
| pBBR111-mamK^RS-1^-Dendra2 | Expression of MamK^RS-1^-Dendra2 in *E. coli*. | This study |
| pBBR111-Dendra2-mad28 | Expression of Dendra2-Mad28 in *E. coli*. | This study |
| pBBR111-Dendra2-mamK^RS-1^ | Expression of Dendra2- MamK^RS-1^ in *E. coli*. | This study |
| pBBR111-∆1-30mad28-Dendra2 | Expression of ∆1-30Mad28-Dendra2 in *E. coli* and AMB-1. | This study |
| pBBR111-∆1-48mad28-Dendra2 | Expression of ∆1-48Mad28-Dendra2 in *E. coli* and AMB-1. | This study |
| pBBR111-∆1-90mad28-Dendra2 | Expression of ∆1-90Mad28-Dendra2 in *E. coli* and AMB-1. | This study |

Table S2 The primers used in this study.

| **Primer** | **Sequence** | **Description** |
| --- | --- | --- |
| mad28_RS-1_f | GAAGGAGATATACATATGTCACAAGAAGATAAGGC | *mad28* fragment upstream |
| His-insert-R | GATGGCGCTGACAGAAGCAGACAATTCGAGCTCCGTC | *mad28* fragment downstream |
| pET29_r | GTTTAACTTTAAGAAGGAGATATACAT | pET29b linearization upstream |
| His-linear-F | AATTCGAGCTCCGTCGACAAG | pET29b linearization downstream |
| Mad28f1 | GAAGGAGGACTCGAGATGTCACAAGAAGATAAGGCC | mad28 fragment upstream |
| Mad28r1 | GGCGCTGACAGAAGCAGACTAAGAATTCCTGCAGCCC | mad28 fragment downstream |
| MamKRS1_f1 | GAAGGAGGACTCGAGATGAGTGGTTCGAATGTGCTC | *mamK^RS-1^* fragment upstream |
| MamKRS1_r1 | GGGATGAACTGCGCAAAGACTAAGAATTCCTGCAGCCC | *mamK^RS-1^* fragment downstream |
| pBBR_CG_f1 | GAATTCCTGCAGCCCGGGGGATCC | pBBR_mamC-gfp/mamK^AMB-1^ linealization downstream |
| pBBR_CG_r1 | GCTGTACAAGTGAAGGAGGACTCGAG | pBBR_mamC-gfp/mamK^AMB-1^ linealization upstream |
| crescentin forward primer | TAAGAAGGAGATATACCATGCGCCTGCTCTCG | *crescentin* fragment upstream |
| crescentin reverse primer | CTGCGTGAGGATCCGGCTGCTAA | *crescentin* fragment downstream |
| pET15b forward primer | GGATCCGGCTGCTAACAAAGCCCGAAAGG | pET15b linearization downstream |
| pET15b reverse primer | GTTTAACTTTAAGAAGGAGATATACC | pET15b linearization upstream |
| C terminal 28_F | ACAGGAGGACTCGAGATGTCACAAGAAGATAAGGC | *mad28* fragment upstream |
| C terminal 28_R | GATGGCGCTGACAGAAGCAGACGGTACCTTAAGATCT | *mad28* fragment downstream |
| K_RS1_Den_f2 | ACAGGAGGACTCGAGATGAGTGGTTCGAATGTGCTC | *mamK^RS-1^* fragment upstream |
| K_RS1_Den_r2 | CTGGGATGAACTGCGCAAAGACGGTACCTTAAGATCT | *mamK^RS-1^* fragment downstream |
| N terminal linker forward | GGTACCTTAAGATCTCGAGCTCCGGAAT | linker fragment upstream |
| N terminal linker reverse | CGATGGCGGCCGCGGATCCT | linker fragment downstream |
| N terminal Dd2 forward | GCGGCCGCGGATCCTATGAATACCCCCGGGATCAAC | *Dendra2* fragment upstream |
| 28Dd2_r | GCCGTCGCAGGTGTGGTGATGAGAATTCCTGCAG | *Dendra2* fragment downstream |
| N terminal reverse | CAGGAAACAGGAGGACTCGAG | pBBR111 linearization upstream |
| pBBRlinear_f | TGAGAATTCCTGCAGCCCGG | pBBR111 linearization downstream |
| N terminal 28_F | GCGGCCGCGGATCCTATGTCACAAGAAGATAAGGC | *mad28* fragment upstream |
| N terminal 28_R | CGCTGACAGAAGCAGACTAAGAATTCCTGCAGCCC | *mad28* fragment downstream |
| Den-K_RS-1_f2 | GCGGCCGCGGATCCTATGAGTGGTTCGAATGTGCTC | *mamK^RS-1^* fragment upstream |
| Den-K_RS-1_r2 | GGGATGAACTGCGCAAAGACTAAGAATTCCTGCAGCCC | *mamK^RS-1^* fragment downstream |
| C terminal Dd2 forward | ACAGGAGGACTCGAGATGAATACCCCCGGGATCAAC | *Dendra2* fragment upstream |
| C terminal Dd2 reverse | CGTTGCCGTCGCAGGTGTGGGGTACCTTAAGATCTC | *Dendra2* fragment downstream |
| Den-K_RS-1_f1 | GAATTCCTGCAGCCCGGGGGATCC | pBBR111 linearization downstream |
| delta1-30-F | ATGGGGTCACTGCCGGAAG | ∆1-30mad28 linearization downstream |
| delta1-48-F | ATGCCGGCGATTGGCCATC | ∆1-48mad28 linearization downstream |
| delta1-90-F | ATGGCGAATCAGCTGAATGC | ∆1-90mad28 linearization downstream |
| N terminal reverse | CAGGAAACAGGAGGACTCGAG | truncated mad28 linearization upstream |

**References**

1. Philippe N, Wu LF. 2010. An MCP-like protein interacts with the MamK cytoskeleton and is involved in magnetotaxis in *Magnetospirillum magneticum* AMB-1. J Mol Biol 400:309-22. https://doi.org/10.1016/j.jmb.2010.05.011

2. Taoka A, Kiyokawa A, Uesugi C, Kikuchi Y, Oestreicher Z, Morii K, Eguchi Y, Fukumori Y. 2017. Tethered magnets are the key to magnetotaxis: direct observations of *Magnetospirillum magneticum* AMB-1 show that MamK distributes magnetosome organelles equally to daughter cells. mBio 8:e00679-17. https://doi.org/10.1128/mbio.00679-17

3. Komeili A, Vali H, Beveridge TJ, Newman DK. 2004. Magnetosome vesicles are present before magnetite formation, and MamA is required for their activation. Proc Natl Acad Sci U S A 101:3839-44. https://doi.org/10.1073/pnas.0400391101
